# The Effect of Low Dose Iron and Zinc Intake on Child Micronutrient Status and Development during the First 1000 Days of Life: A Systematic Review and Meta-Analysis

**DOI:** 10.3390/nu8120773

**Published:** 2016-11-30

**Authors:** Nicolai Petry, Ibironke Olofin, Erick Boy, Moira Donahue Angel, Fabian Rohner

**Affiliations:** 1 GroundWork, Fläsch 7306, Switzerland; ioo523@mail.harvard.edu (I.O.); fabian@groundworkhealth.org (F.R.); 2 Harvest Plus, International Food Policy Research Institute, Washington, DC 20006-1002, USA; E.Boy@cgiar.org (E.B.); m.angel@cgiar.org (M.D.A.)

**Keywords:** Iron, zinc, iron status, zinc status, 1000 days window, infant and young child nutrition, fortification, biofortification

## Abstract

Adequate supply of micronutrients during the first 1000 days is essential for normal development and healthy life. We aimed to investigate if interventions administering dietary doses up to the recommended nutrient intake (RNI) of iron and zinc within the window from conception to age 2 years have the potential to influence nutritional status and development of children. To address this objective, a systematic review and meta-analysis of randomized and quasi-randomized fortification, biofortification, and supplementation trials in women (pregnant and lactating) and children (6–23 months) delivering iron or zinc in doses up to the recommended nutrient intake (RNI) levels was conducted. Supplying iron or zinc during pregnancy had no effects on birth outcomes. There were limited or no data on the effects of iron/zinc during pregnancy and lactation on child iron/zinc status, growth, morbidity, and psychomotor and mental development. Delivering up to 15 mg iron/day during infancy increased mean hemoglobin by 4 g/L (*p* < 0.001) and mean serum ferritin concentration by 17.6 µg/L (*p* < 0.001) and reduced the risk for anemia by 41% (*p* < 0.001), iron deficiency by 78% (ID; *p* < 0.001) and iron deficiency anemia by 80% (IDA; *p* < 0.001), but had no effect on growth or psychomotor development. Providing up to 10 mg of additional zinc during infancy increased plasma zinc concentration by 2.03 µmol/L (*p* < 0.001) and reduced the risk of zinc deficiency by 47% (*p* < 0.001). Further, we observed positive effects on child weight for age *z*-score (WAZ) (*p* < 0.05), weight for height *z*-score (WHZ) (*p* < 0.05), but not on height for age *z*-score (HAZ) or the risk for stunting, wasting, and underweight. There are no studies covering the full 1000 days window and the effects of iron and zinc delivered during pregnancy and lactation on child outcomes are ambiguous, but low dose daily iron and zinc use during 6–23 months of age has a positive effect on child iron and zinc status.

## 1. Introduction

Globally iron and zinc deficiencies are among the most widespread micronutrient deficiencies. While people of all ages are at risk, children and women of reproductive age are at elevated risk of experiencing concurrent deficiencies, especially in low-income countries [1,2]. Even mild deficiencies of one or both nutrients may contribute to increased morbidity and mortality [1]. The first 1000 days of life—the period from conception to the child’s 2nd birthday—are most crucial, since some developmental and functional delays during this period are either irreversible or only partly reversible [3]. In utero exposure to iron deficiency has been associated with impaired brain maturation of the fetus [4,5], while during infancy and childhood, iron deficiency could lead to impaired cognitive and physical functionality and increased risk of mortality [1,6]. Zinc is essential for cellular differentiation and maturation and maternal zinc deficiency could lead to growth retardation and other developmental defects of the fetus [7]. Further, zinc deficient infants and young children are prone to infections and growth retardation [8].

The major cause of micronutrient malnutrition is a diet consisting mainly of staple foods and lacking in animal sources [9]. When compounded by exposure to environments laden with pathogens, there is a synergistic worsening of the malnutrition burden [10,11]. Although high dose supplementation has been successful in reducing the prevalence of micronutrient deficiencies, programs often only inadequately reach rural or marginalized populations; also high dose iron supplementation has been exposed to criticism in the past decade in malaria endemic areas [12]. Other interventions such as fortification and biofortification, delivering smaller amounts of micronutrients on a daily basis, might be more effective in reducing the prevalence of micronutrient deficiencies in populations at risk; yet, their effect on the infant during the first 1000 days of life starting from pregnancy until 23 months of age has hardly been investigated.

The objective of this work was thus to evaluate the potential of interventions delivering daily doses of iron and zinc in concentrations up to approximately the Recommended Nutrient Intake (RNI) in diets with low bioavailability [1] during the first 1000 days of life on child micronutrient status and health.

For this we used data from randomized and quasi-randomized trials of fortification, supplementation or biofortification interventions, in which iron and zinc were provided more than three times a week in concentrations up to approximately the (RNI) for women (iron: 45 mg/day; zinc 20 mg/day) and children 6–23 months (iron: 15 mg/day; zinc: 10 mg/day).

## 2. Materials and Methods

### 2.1. Search Strategy

We searched the WHO e-Library of Evidence for Nutrition Actions, the Cochrane Central library, Web of Science, and MEDLINE/PUBMED databases to identify systematic reviews and meta-analyses that investigated the effects of iron and zinc interventions (fortification, supplementation, or biofortification interventions) on nutritional, developmental and health outcomes of children. We restricted the search to reviews published from 2005 to 2015. The following search strategy was adapted for each database: (Fortification OR biofortification OR supplementation) AND (iron OR zinc OR multiple micronutrients OR micronutrient powder) AND (women OR children OR infants OR toddlers). For the relevant topics, 82 potentially useful reviews and meta-analyses published from 2005 to 2015 were identified, the most recent ones conducting their literature searches up until 2014. We searched their reference lists for suitable original studies. Additional searches for original studies published between 2011 and October 2015 were conducted in Web of Science and MEDLINE/PubMed using the following strategies for each age group of interest:

(a) Children: (Biofortification OR fortification OR supplementation) AND (iron OR zinc OR micronutrient powder OR multiple micronutrients) AND (children OR infants OR toddlers) AND (trial OR study OR survey OR assessment).
(b) Pregnant women: (Biofortification OR fortification OR supplementation) AND (pregnant women OR lactating women OR maternal) AND (iron OR zinc OR micronutrient powder OR multiple micronutrients) AND (trial OR study OR survey OR assessment).
(c) Lactating women: (Biofortification OR fortification OR supplementation) AND lactating women AND (zinc OR iron OR micronutrient powder OR multiple micronutrients OR breast milk) AND (trial OR study OR survey OR assessment).

### 2.2. Inclusion and Exclusion Criteria

Types of trials and interventions: Randomized controlled trials (RCTs) and quasi-experimental studies were included in the review. Studies were eligible if they assessed the impact of iron or zinc supplementation, fortification or biofortification interventions on the micronutrient status, growth or health outcomes of children (details below). Only studies where the daily iron and zinc dose did not exceed 15 mg and 10 mg, respectively for children and 45 mg and 21 mg, respectively for women, were included. We considered micronutrient powders and crushable tablets (foodlets) as fortification, since they are consumed as part of a normal meal. Furthermore, we included studies investigating the effects of iron or zinc supplements, as long as they were within the dose range we specified. We defined supplements as compounds, which are routinely consumed separately from a normal meal, including tablets, pills, drops, capsules, syrups, drinks, biscuits, and lipid-based supplement (LNS). Only studies administering the micronutrients >3 times a week were included.

Types of participants: For interventions administered to the mother, we included studies that provided interventions to pregnant women or lactating women, regardless of their health status. For interventions administered to children, we were interested in effects on young children, thus only studies where over 50% of participating children were 6–23 months old were included. Only studies involving apparently healthy children were considered, with the exception that studies including malnourished (underweight, stunted, wasted) children and children suffering from anemia or deficiencies of iron and zinc were included. We did not include therapeutic studies, such as short term zinc supplementation studies to treat acute diarrhea.

### 2.3. Study Design and Comparison Groups

The control groups of the included fortification trials either received unfortified foods or regular diets. Studies were also included if both the control and intervention groups received the same fortified foods, but either with different iron or zinc concentrations, or if the control group received an identical micronutrient compound given to the intervention group, but without iron or zinc. For supplementation, the control groups of the included trials either received no supplements, placebo, a lower concentration of iron or zinc or different micronutrients identical to intervention group preparations, except that they excluded zinc and/or iron.

Studies were only included in meta-analyses if the data for outcomes of interest were presented in a manner that allowed inclusion in the meta-analysis (i.e., data could only be used when presented as mean (SD or SE), mean (95% CI), median (95% CI), or median (range), but not when reported as median (IQR)). Where studies did not report on the average daily micronutrient intake, but instead stated the micronutrient concentration per 100 mL/100 g or the daily micronutrient intake in mg/kg body weight, we calculated the average intake as appropriate.

### 2.4. Outcome Measures

Only child outcomes were of interest, even when interventions were administered to pregnant or lactating women. For example, with regard to effects of prenatal iron supplementation on anemia, maternal anemia was not the focus of this analysis; rather, the infant/child anemia was of relevance even if the mother received the intervention.

Outcomes evaluated include: (1) hemoglobin (Hb) concentration (g/dL); (2) anemia (%; defined as Hb <110 g/L); (3) serum ferritin concentration (µg/L); (4) iron deficiency (%; defined as serum ferritin <10 µg/L or <12 µg/L); (5) iron deficiency anemia (%; defined as hb <105 g/L or <110 g/L and serum ferritin <10 µg/L or <12 µg/L); (6) serum or plasma zinc (µmol/L); (7) zinc deficiency (%; defined as serum zinc <10.7 µmol/L); (8) birth outcomes (birth weight in g; prevalence of low birth weight in %, defined as weight <2500 g); (9) infant anthropometric measures (height for age *z*-score (HAZ); weight for age *z*-score (WAZ); weight for height *z*-score (WHZ); stunting (≤−2 HAZ scores), wasting (≤−2 WHZ scores) underweight (≤−2 WAZ scores)); (10) mental and motor development (Bayley mental development index (MDI); Bayley psychomotor development index (PDI)); (11) morbidity (diarrhea, fever and respiratory infection).

### 2.5. Data Synthesis and Statistical Analysis

From each eligible study, we extracted all data that would allow the estimation of the effects of interest. For example, for zinc studies, data were extracted for groups assigned to zinc alone compared with placebo, as well as data from groups assigned to more than one nutrient including zinc compared with a group assigned to the identical nutrient combination but excluding zinc. Where the total number of comparisons was sufficiently large, we examined the influence of combining such comparisons into one meta-analysis, and where possible, conducted sub-group analyses examining the possibility of nutrient interactions. Additionally, where studies had more than one group assigned to the nutrient of interest (for instance, different nutrient dosages) but only one suitable comparison group, we included such data as separate estimates for the meta-analysis. For that, we divided the comparison group into two groups (or more, when required) with smaller sample sizes, so the same children were not involved in more than one comparison. To examine if this decision affected the results, we conducted sensitivity analyses combining all relevant control groups of affected studies into a single comparison group, and combining all relevant intervention groups into a single group, for some outcomes. As results were very similar, we chose to continue analyses with the sub-groups. When studies reported outcomes at multiple time points, we selected only the results reported at the study end (or the latest time point) for the meta-analyses. Most studies were individually randomized trials, but where cluster randomized trials were eligible for inclusion, we estimated design effects to adjust for the influence of intra-cluster correlations on the precision of estimates as appropriate. To maximize the number of studies that could contribute to meta-analyses of continuous outcomes, we converted medians (reported with ranges) and geometric means (reported with standard deviations, standard errors or confidence intervals) to arithmetic means and standard deviations using methods developed by Hozo et al. [13] and Higgins et al. [14] and examined the effect that combining transformed and untransformed estimates had on pooled results. Studies were excluded from meta-analyses if published reports presented insufficient information for estimating desired effect estimates and variances.

For continuous outcomes, we estimated pooled mean differences or standardized mean differences and confidence intervals, as appropriate, while for categorical outcomes, we estimated pooled risk ratios and confidence intervals. Study-level effect estimates were pooled using the random effects meta-analysis method by DerSimonian and Laird [15]. We assessed heterogeneity among studies, and used the method proposed by Higgins et al. to measure the inconsistency (*I*^2^) of effect estimates across studies [16]. Heterogeneity among study estimates was considered to be substantial if the *I*^2^ exceeded 50%. For outcomes having at least 10 comparisons per variable of interest, we explored sources of heterogeneity by conducting pre-specified sub-group analyses and meta-regressions, to examine whether effects were modified by the study-level factors: micronutrient doses provided, type of intervention (whether fortification or supplementation), study quality and baseline micronutrient status. Funnel plots were constructed for visual assessment of the variability of individual study estimates and to evaluate the possibility of publication bias, and when appropriate, Egger’s tests were used to examine if effect estimates varied with study sample size.

All analyses were conducted using the metafor package [17] of the R statistical program (R version 3.1.3 (2015-03-09), The R Foundation, Vienna, Austria, 2015).

### 2.6. Assessment of Quality and Risk of Bias

We assessed study quality in three areas—random sequence generation, adequacy of blinding of study participants and personnel and completeness of outcomes assessment—but did not exclude studies based on the assessment of quality. Studies were categorized as being of the ‘highest quality’ if interventions were randomly assigned, both participants and study personnel were adequately blinded to the intervention assignment, and if outcomes were assessed in at least 75% of the enrolled study population. Intermediate quality trials were randomized trials for which only one of the remaining two criteria was suboptimal, lowest quality trials were randomized trials for which both the remaining criteria were suboptimal, and quasi-experimental trials (although rarely included) were given the very lowest rating. The effect of pooling results from studies of different quality was examined in sub-group analyses where possible.

The quality of the evidence resulting from each analysis was assessed using the Grading of Recommendations Assessment, Development, and Evaluation (GRADE) method [18]. Domains included: risk of bias within studies (internal validity), inconsistency or heterogeneity of results across studies, indirectness or use of proxy populations, interventions or outcomes measures, imprecision (large variability and wide confidence intervals), number of studies and risk of publication bias. The evidence was judged as high quality if further research studies were unlikely to change the pooled estimate obtained; moderate quality if further research could alter the current estimate; low quality if further research was needed to confirm the magnitude and direction of the true effect; and very low quality if there was great uncertainty about the validity of the pooled estimate. It should be noted that because the GRADE method considers several domains and not just internal study validity, confidence in pooled estimates could be ‘low’ or ‘very low’ even if all studies that contributed to the estimate were well conducted.

## 3. Results

### 3.1. Literature Search

We identified and screened 4542 records, reviewed 326 full-texts for eligibility and finally included 90 studies in the review (Figure S1). Identified studies were supplementation or fortification studies—no biofortification studies qualified for inclusion. Thirty-three iron intervention studies and 47 zinc intervention studies involving children 6–23 months old met our inclusion criteria. Of the prenatal intervention studies that reported child outcomes, seven iron studies and 10 zinc studies met our inclusion criteria. Additionally, we identified one eligible study that delivered zinc to lactating women. The major reasons for excluding studies were: (a) micronutrient doses higher than our selected threshold; (b) ineligible ages, e.g., more than half of participating children over 23 months old and (c) dosing frequencies less than our threshold.

### 3.2. Effects of Low-Dose Iron Interventions during Pregnancy and Lactation on Child Outcomes

Six studies reported birth weights of infants [19,20,21,22,23,24] while five studies reported on the effects of iron on the prevalence of low birth weight (Table 1; [19,21,22,23,24]). Exclusively supplementation trials contributed to the analyses, delivering daily 18–30 mg of iron. We found that low dose iron interventions during pregnancy do not significantly change birth weights (*p* = 0.17; Figure S2) or the prevalence of low birth weight (*p* = 0.23; Figure S3) among the offspring. We did not identify any studies investigating the effects of prenatal iron interventions on child micronutrient status or child growth within the first two years of life. We identified a study by Li and colleagues [25] that investigated the effects of prenatal iron and folic acid compared with folic acid alone on PDI and MDI scores of children at 3, 6, and 12 months of age. No differences were detected between the iron/folic acid group and the folic acid only group. The main results of the meta-analysis of prenatal iron interventions are summarized in Table 1.

### 3.3. Iron Interventions in Children 6–23 Months of Age

The results of meta- analyses of iron intervention studies involving children are presented below and summarized in Table 2 and Table 3.

*Effect on hemoglobin concentration:* We identified 30 RCTs [26,27,28,29,30,31,32,33,34,35,36,37,38,39,40,41,42,43,44,45,46,47,48,49,50,51,52,53,54,55], contributing 43 comparisons, for evaluating the effect of up to 15 mg of additional iron daily on hemoglobin levels of children 6–23 months of age. In total, 6569 children contributed to the pooled estimate. The iron interventions led to significantly higher hemoglobin concentrations in children, compared with no iron (pooled mean difference 4.07 g/L (95% CI: 2.82, 5.33; Figure S4), although there was significant heterogeneity of results (*I*^2^ 82.5%; *p* < 0.0001). To examine the heterogeneity, we conducted meta-regression analyses and sub-group analyses investigating whether effect sizes were different for pre-specified sub-groups defined by the intervention dose (Figure S5), type of intervention (fortification vs. supplementation; Figure S6) and study quality (Figure S7). The increases in hemoglobin concentrations resulting from the intervention were significantly higher for supplementation trials than fortification trials (*p* < 0.01) and significantly lower as study quality worsened (*p* < 0.05). There was no significant difference in effect sizes by intervention dose (*p* = 0.12). Adjusting for these variables simultaneously did not substantially explain the observed heterogeneity (residual *I*^2^ 81%, *p* < 0.0001).

*Effect on anemia prevalence:* 22 RCTs involving a total of 5647 children contributed [26,27,29,30,31,32,34,35,36,37,38,39,43,44,45,47,48,51,52,54,55,56] to the meta-analysis. The iron interventions resulted in a 41% reduction in children’s risk of anemia compared with no iron (pooled relative risk (RR) 0.59 (95% CI: 0.49, 0.70), Figure S8), although there was considerable heterogeneity of results, *I*^2^: 73.8% (*p* value 0.0008). We investigated the influence of the intervention dose on effect sizes (Figure S9) and while it appears that interventions delivering 6–8 mg and >8–10 mg iron per day reduce the risk of anemia by 46% and 41%, respectively, intervention dose did not significantly explain the differences in study effect sizes (*p* = 0.32). This agrees with the results of sub-group analyses for hemoglobin outcomes, where the largest effect sizes were observed for iron interventions delivering between 6 mg and 10 mg per day (Figure S5).

*Effect on serum ferritin:* Twenty-one RCTs [26,27,28,31,32,33,34,36,37,38,40,42,44,45,48,49,50,51,52,53,54], contributing 25 comparisons, provided sufficient data for the meta-analysis of the effect of low dose iron interventions on serum ferritin concentrations in young children. In total, 4291 children contributed to the pooled estimate. After pooling the results, the iron intervention resulted in significantly higher serum ferritin concentrations compared with controls (mean difference 17.3 µg/L (95% CI: 13.1, 21.2; Figure S10), although there was significant heterogeneity, *I*^2^ 95.1% (*p* < 0.001). To understand the substantial heterogeneity in the results, we conducted meta-regression analyses and sub-group analyses investigating the influence of the dose (Figure S11), type of intervention (fortification vs. supplementation; Figure S12) and quality of interventions (Figure S13) on the effect size estimates. The effect sizes resulting from the intervention were significantly higher for higher doses (*p* < 0.01) and for supplementation trials compared with fortification trials (*p* < 0.001). Meta-regression results suggested that effects diminished as study quality worsened, although not significantly (*p* = 0.08). However, adjusting for dose, study quality, and intervention type simultaneously did not explain much of the observed heterogeneity (residual *I*^2^ 88%) and the change in effect size associated with increasing doses was no longer significant after adjusting for the quality of studies and type of intervention. We further examined the effect of baseline iron deficiency (categorized as low prevalence if <15% and high prevalence if ≥15%) on serum ferritin results. Only nine comparisons had information on baseline iron status. The pooled mean difference in serum ferritin for comparisons where baseline iron deficiency was high was not considerably different from the comparisons where baseline iron deficiency was low (Figure S14), and baseline iron deficiency did not explain the heterogeneity in serum ferritin results *(p* value = 0.76). Yet, with the limited number of studies, inadequate power could be a problem. In order to maximize the number of studies in the sub-group analysis, we created an ‘in-house’ measure of baseline iron status: from all reported mean serum ferritin concentrations, we took the 50th percentile (29.2 ug/L). Studies were categorized as having low iron status if mean serum ferritin was <29.2 and adequate if mean serum ferritin ≥29.2. This variable could be created for 21 comparisons. However, even so, we did not detect a significant effect of baseline iron status and it did not account for the observed heterogeneity of serum ferritin results (Figure S15; *p* value = 0.99). The created variable may be an inadequate surrogate for participants’ baseline iron deficiency levels.

*Effect on prevalence of ID and IDA:* Thirteen trials [26,29,31,32,34,36,38,39,43,45,48,52,54] and eight trials [26,29,34,35,36,40,43,48] conducted mainly in Asia, provided 17 and 13 comparisons, respectively, that could be included in meta-analyses investigating the effect of the iron interventions on the risk of ID and IDA respectively among children 6–23 months old. The majority of trials defined ID as serum ferritin concentrations <12 µg/L or <10 µg/L while IDA was mainly defined as hemoglobin <110 g/L with serum ferritin concentrations <12 µg/L. The pooled relative risk from the random effects meta-analysis was 0.22 (95% CI: 0.14, 0.35), *I*^2^: 86.3% (Figure S16) for ID and 0.20 (95% CI: 0.11, 0.37), *I*^2^: 64.2% (Figure S17) for IDA, meaning that providing children 6–23 months old with up to 15 mg of iron daily significantly reduced their risk of ID and IDA by 78% (95% CI: 65% to 86% reduction) and 80% (95% CI: 63% to 89% reduction) respectively.

*Effect on growth:* Ten RCTs examined the effect of iron on WAZ [35,36,37,38,41,43,44,48,52]; nine studies each contributed to the meta-analyses of the iron effect on WHZ [35,36,37,38,42,43,44,48,52] and HAZ [35,36,37,38,41,43,44,48,52]. There was no significant effect of iron on WAZ (Figure S18; Table 3; *p* = 0.69), WHZ (Figure S19; Table 3; *p* = 0.62) or HAZ (Figure S20; Table 3; *p* = 0.59). Similarly, random effects meta-analysis indicated (Figures S21 and S22; Table 3) that there was no effect of iron on the risk for stunting [35,36,37,48] or wasting [35,36,37,48].

*Effect on diarrhea, fever and respiratory infection:* We identified eight eligible studies [27,38,42,44,48,49,57,58], with a total of 11 comparisons, reporting on the impact of iron use on diarrhea, respiratory infection and/or fever. Given the variability of methods and outcome measures in the studies reviewed, it was not possible to conduct a meta-analysis without introducing a selection bias. The effect of iron alone was compared to placebo in seven RCTs, three compared the effect of iron and zinc to zinc alone, and one study compared the effect of MNP with and without iron on morbidity. Seven studies looked at the impact of iron on diarrhea, seven on respiratory infection and five on fever. None of the studies reported a beneficial effect of iron on any of the morbidities.

*Effect on mental and motor development:* Four eligible studies (five comparisons) reported children’s Bayley mental development index (MDI; [27,36,51,54]) and Bayley psychomotor development index (PDI; [27,36,51,54]) scores. Children included in the analysis received daily 5–10 mg fortification or supplementation iron for 3–9 months. The analyses indicated that interventions delivering dietary doses of iron have no effect on mental development (*p* = 0.6; Figure S23; Table 3) and psychomotor development scores (*p* = 0.5; Figure S24; Table 3).

### 3.4. Zinc Interventions during Pregnancy and Lactation

Few data on the effect of maternal supplementation on infant outcomes are available during pregnancy and the lactation period for most of the outcomes of interest and thus a meta-analysis was only conducted for birth outcomes. Results of single studies investigating growth and micronutrient status are discussed in the section below.

#### 3.4.1. Effect on Birth Weight and Prevalence of Low Birth Weight

The results of meta- analyses of studies conducted in pregnant women are summarized in Table 4.

Eight studies [59,60,61,62,63,64,65,66] and nine comparisons contributed to the meta-analysis for the effect of prenatal zinc use on birth weight, and six studies (seven comparisons) contributed to the meta-analysis of prenatal zinc and low birth weight prevalence [59,61,62,63,65,67]. We found that delivering up to 21 mg of zinc daily during pregnancy has no significant effect on birth weights of offspring (*p* = 0.94; Figure S25) or the prevalence of low birth weight (*p* = 0.83; Figure S26).

#### 3.4.2. Effect on Infant Growth and Micronutrient Status

Two studies examining child growth outcomes and one study examining the zinc status of children were identified. Prawirohartono et al. [60] looked at growth of infants whose mothers received either 20 mg zinc only, vitamin A only, 20 mg zinc and vitamin A or placebo during pregnancy. They monitored growth until 23 months of age. Zinc had a significant beneficial effect on HAZ at 6 months. No effects on any other growth parameters at any time point were observed. Iannotti et al. [68] administered 15 mg zinc daily to pregnant women from gestational week 16 until 1 month after delivery and measured infant growth from birth to 12 months of age. They did not assess any predefined outcomes, but observed differences between intervention and control group in weight, calf, chest, mid-upper arm circumferences, and skinfold thicknesses from age 6 month to 12 month; and no effect on length or head circumference.

Caulfield and colleagues [59] looked at the zinc status of neonates after zinc supplementation of pregnant women. Women were included at gestational ages of 10–24 weeks and received a daily dose of 15 mg zinc. They reported a significantly higher cord blood zinc concentration in neonates whose mothers received zinc supplementation, compared with controls.

Only one study was identified that investigated the effects of maternal zinc supplementation/fortification during the lactation period on infant outcomes. The effect of maternal zinc supplementation on serum zinc concentration and growth in children after birth to 9 months of age was investigated by Salmenpera et al. [69]. They gave two different zinc doses (20 mg, 40 mg daily) to lactating women and compared the impact to a control group. Breast milk zinc concentration did not differ between groups at 0, 2, and 4 months after delivery, but was higher at 6 and 7.5 months in the group receiving 40 mg/day of zinc. When analyses were restricted to exclusively breastfed infants, no difference in serum zinc concentration was detected between intervention and control groups at any time point and maternal zinc supplementation had no effect on infant growth.

### 3.5. Zinc Interventions in Children 6–23 Months of Age

#### 3.5.1. Effect on Serum or Plasma Zinc Concentrations and Zinc Deficiency

The main results of the meta-analyses of studies in children investigating the effect of zinc administration on serum zinc and zinc status are presented in Table 5.

Twenty-three [36,43,48,59,70,71,72,73,74,75,76,77,78,79,80,81,82,83,84,85,86,87,88] RCTs (contributing 35 comparisons) provided sufficient information to be included in the meta-analysis summarizing the effect of up to 10 mg of additional zinc daily on the serum or plasma zinc concentrations of children 6–23 months old.

The pooled estimate suggests that the zinc interventions significantly increased serum or plasma zinc concentrations by 2.03 μmol/L compared with no zinc (95% CI 1.21, 2.85 µmol/L; *p* < 0.0001, Figure S27), but with significant heterogeneity (*I*^2^ 98%). Meta-regression analyses suggest that effect sizes were larger at higher doses (*p* < 0.05, Figure S28), and for supplementation compared with fortification trials (*p* = 0.05, Figure S29), while study quality did not significantly explain the heterogeneity. Dose, intervention type and study quality together did little to explain the heterogeneity of findings (residual *I*^2^ 97.9%). Subgroup analyses stratified by dose suggested that the strongest effect on serum zinc was obtained in studies delivering daily 7–10 mg (3.0 µmol/L; (95% CI 1.51, 4.48)). A small but significant effect was also detected in studies administering 4–<7 mg/day (0.9 µmol/L (95% CI 0.08, 1.71; Figure S28)). Stratification by intervention type revealed that the pooled effect size of fortification trials on serum zinc concentrations was small and not statistically significant (0.31 µmol/L (95% CI −0.12, 0.75; Figure S29), whereas supplementation showed a larger and significant difference (2.07 µmol/L (95% CI 1.5, 3.4).

Baseline zinc deficiency levels were reported for 12 comparisons with serum zinc as the outcome. We categorized baseline zinc deficiency as low prevalence if <25% and high prevalence if ≥25%. The pooled mean difference in serum zinc was similar regardless of baseline zinc deficiency status (Figure S30), and baseline zinc deficiency explains 8.4% of the heterogeneity in serum zinc results (*p* value = 0.15). In order to maximize the number of studies in the sub-group analysis, we created a measure of baseline zinc status: from all reported serum zinc means, we took the 50th percentile (10.75 μmol/L). Studies were categorized as having low zinc status if mean serum zinc was <10.75 μmol/L and adequate if mean serum zinc ≥10.75. This variable could be created for 25 comparisons. It did not explain any of the observed heterogeneity of serum zinc results (*p* value = 0.96; Figure S32). The created variable may be an inadequate surrogate for participants’ baseline zinc deficiency levels.

For the effect of zinc interventions on the risk of zinc deficiency among children 6–23 months old, 12 RCTs [35,39,43,48,71,72,74,77,78,81,84,87], which provided 22 comparisons, contributed to the meta-analysis. Half defined zinc deficiency as serum or plasma zinc concentrations <10.7 µmol/L, while the others defined it using a cutoff of <~9.9 µmol/L.

The pooled relative risk from the random effects meta-analysis was 0.47 (95% CI 0.32, 0.69), *I*^2^: 92%, meaning that providing children 6–23 months old with up to 10 mg of zinc daily significantly reduced their risk of zinc deficiency by 53% (95% CI 25% to 64% reduction; Figure S32).

#### 3.5.2. Effect on Growth

Results of studies investigating effects of zinc interventions on child growth are summarized in Table 6.

Twenty-one [36,37,43,48,74,75,78,83,84,85,86,88,89,90,91,92,93,94,95,96,97] studies (31 comparisons) contributed to meta-analyses of zinc effects on WAZ, and 20 studies yielding 30 comparisons [36,37,43,48,74,75,78,82,83,84,85,86,88,89,90,91,92,94,95,97] reported HAZ outcomes. The WHZ meta-analysis included 16 studies [36,37,43,48,74,75,78,82,84,85,88,89,90,91,92,97] with 25 comparisons. After pooling the studies, zinc interventions compared with placebo or no zinc slightly but significantly increased WAZ (mean difference: 0.05, 95% CI 0.0, 0.1) and WHZ (mean difference: 0.04, 95% CI 0.0, 0.08), while the HAZ result was not significant (mean difference: 0.00, 95% CI −0.04, 0.03; Figures S33–S35).

Fewer studies categorized children as stunted, wasted or underweight. Six studies with 10 comparisons [36,37,48,74,76,79] were included in the meta-analysis for the effect of zinc interventions on childhood stunting (HAZ <−2), five studies with eight comparisons [37,74,76,79] in the meta-analysis for childhood underweight, and six studies yielding 10 comparisons [36,37,48,74,76,79] in the meta-analysis for childhood wasting.

The zinc interventions were not associated with significant reductions in the risk of child stunting (RR 0.97, 95% CI 0.9, 1.04), wasting (RR 0.98, 95% CI 0.79, 1.21) or underweight (RR 0.99, 95% CI 0.90, 1.09; Figures S36–S38).

#### 3.5.3. Effects on Diarrhea, Fever, and Respiratory Infections

In total, we identified 24 studies [48,57,70,71,73,74,75,76,77,79,82,85,87,88,89,90,91,93,97,98,99,100,101,102,103], with 33 comparisons, meeting our inclusion criteria and reporting on the impact of zinc on diarrhea, respiratory infections and fever. Twenty-one studies were individually randomized and three studies cluster randomized trials. We decided not to do a meta-analysis given the considerable variability of methods and outcome measures in the studies reviewed: more than half of the studies could not have been included in the analysis and thus, results might have been biased.

Similar to previous reviews, we found conflicting results for the effect of zinc intake on diarrhea, but not on respiratory infection and fever prevalence and/or incidence. Of the 16 studies [48,57,70,75,82,85,87,88,91,97,99,100,102,103,104,105] reporting on fever and/or respiratory infections, only 3 [82,91,104] found a positive effect of zinc intake. Out of the 24 studies investigating the effect of zinc on diarrhea, 11 studies [48,57,70,74,75,76,79,85,87,88,99] found no effect at all. Ten [71,73,89,90,91,93,97,100,101,102] reported a positive effect of zinc use on the prevalence and or incidence of diarrhea. Two [82,103] found a significant positive effect of zinc on diarrhea in the stunted sub-group, and Sazawal et al. [77] reported only a significant effect of zinc in children with low serum zinc concentrations and children older than 11 month of age. Age dependency has also been reported by Wuehler et al. [97], with a strong effect in the age group of 11.5–17.4 months and no effect in older children (17.5–30 months).

#### 3.5.4. Effects on Mental and Motor Development

Three [57,106,107] eligible studies reported MDI and PDI scores and found no significant impact of zinc interventions on the two outcomes.

### 3.6. The Interaction of Iron and Zinc in Interventions in Children 6–23 Months of Age

#### 3.6.1. Effect of Zinc on Serum Ferritin

**Figure 1:**
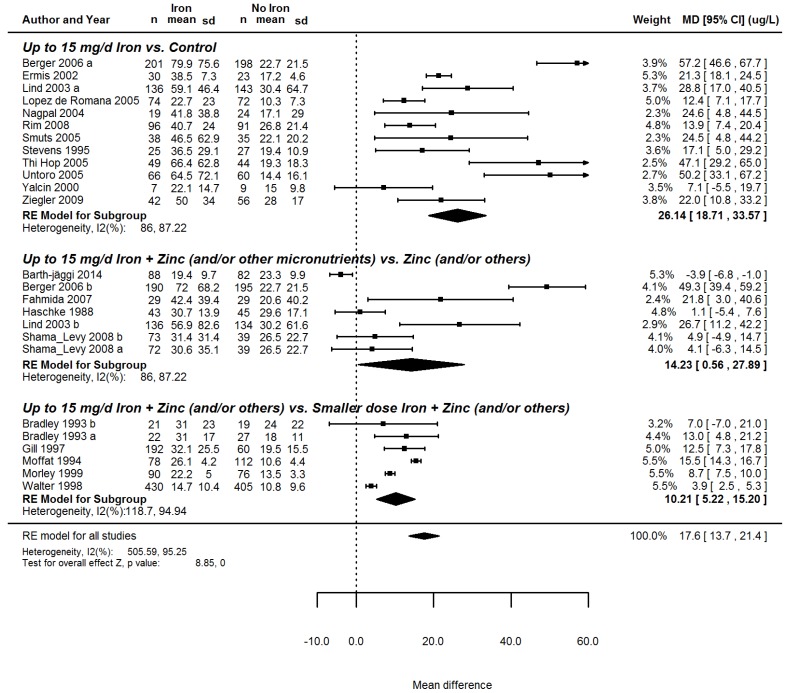
Forest plot summarizing the interactions of iron and zinc, supplying up to 15 mg of additional iron daily to children 6–23 months old on serum ferritin.

Sub-group analyses of the 21 RCTs [26,27,28,31,32,33,34,36,37,38,40,42,44,45,48,49,50,51,52,53,54] reporting on the effect of iron interventions on serum ferritin indicated that iron has the strongest beneficial effect when it is the sole micronutrient administered (mean increase of 26.1 µg/L) and that it has a smaller, albeit significant, effect on serum ferritin when it is administered in combination with zinc (Figure 1).

#### 3.6.2. The Effect of Iron on Serum Zinc

**Figure 2:**
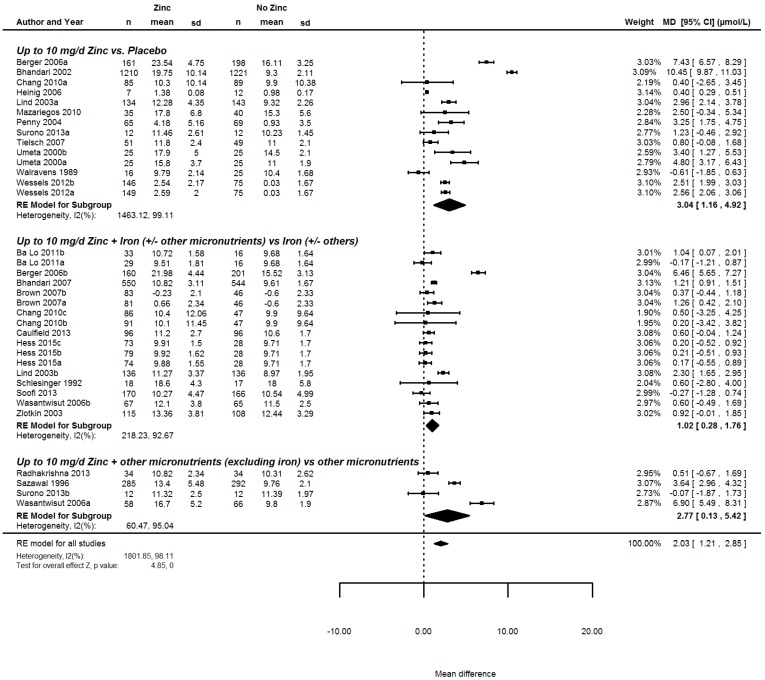
Forest plot summarizing the effect of interventions supplying up to 10 mg of additional zinc daily to children 6–23 months old, on serum or plasma zinc concentrations, stratified by micronutrient composition.

We found 23 studies [36,43,48,59,70,71,72,73,74,75,76,77,78,79,80,81,82,83,84,85,86,87,88], with 35 comparisons, 14 of them comparing zinc alone to placebo, 17 of them comparing zinc in combination with iron to iron only and four of them comparing zinc and other micronutrients to other micronutrients. Sub-group analyses showed that if iron was part of the intervention, the effect of zinc on serum/plasma zinc concentrations was considerably lower (1.02 µmol/L, 95% CI 0.28, 1.76), compared to interventions delivering zinc alone (3.04 µmol/L, 95% CI 1.16, 4.92) and to interventions delivering zinc and other micronutrients, excluding iron (2.77 µmol/L, 95% CI 0.13, 5.42; Figure 2).

### 3.7. Quality of the Evidence across Studies

We used the GRADE approach to assess the quality of evidence across studies (Table S1). We considered that a publication bias or imprecision was unlikely for all outcomes. In contrast, the large heterogeneity, the lack of studies, and indirectness were considered important factors in the quality of evidence across studies. For indirectness of evidence we rated down in case ≥50% of the included studies was not food based interventions. For interventions delivering iron compared to a placebo group, the overall quality of evidence was found to be high for iron status (using serum ferritin concentration), ID and IDA; moderate for hemoglobin, HAZ, WAZ, and WHZ; low for anemia, stunting and wasting, whereas for birth weight, low birth weight, MDI and PDI the quality of evidence was very low. For interventions administering zinc compared to placebo the quality of evidence was moderate for birth weight, prevalence of low birth weight, zinc deficiency, zinc status (using serum zinc concentration), stunting, wasting, HAZ, WAZ, and WHZ, and low for underweight.

## 4. Discussion

We found that interventions delivering iron and zinc in concentrations up to the RNI during the 1000 days window from the prenatal period to the first 2 years of a child’s life can have positive impacts on the iron and zinc status of young children. In particular, when interventions were conducted in children aged 6–23 months old, their hemoglobin levels, and iron and zinc status improved. For both nutrients, supplementation triggered a significantly stronger response than fortification even when doses were similar. For other outcomes assessed—birth weight, growth in childhood, psychomotor development—findings are less conclusive, partly due to the limited number of studies.

We show that low dose iron and zinc interventions providing no more than 45 mg/day of iron and 20 mg/day of zinc during pregnancy may not have an impact on children’s birth weights and prevalence of low birth weight. Similar to our results, other meta-analyses found no effect of zinc on birth weight and prevalence of low birth weight [108,109]. Most of the studies included in those analyses did not meet our inclusion criteria because they gave higher doses of zinc than we were interested in. For iron, our results are also in accordance with a recent meta-analysis, which only observed beneficial effects at iron doses exceeding our threshold [110]. However, due to the scarcity of studies having follow-up data extending beyond the perinatal period, it is unclear if any growth advantages of routine iron and zinc ingestion during pregnancy could manifest later in infancy or early childhood.

We found that the use of iron in dietary doses (no more than 15 mg/day) during infancy and early childhood had positive effects on hemoglobin levels, anemia prevalence, and iron status (serum ferritin, ID and IDA prevalence), which agrees with a systematic review published in 2012 assessing the effect of micronutrient-fortified milk and cereal foods on infants and children [111] and a meta-analysis of supplementation trials in children 6–23 month of age, published in 2013 [112]. We did not find an effect of iron interventions on child growth or morbidity, suggesting that food-based iron interventions at doses investigated here have no beneficial impact on the occurrence of childhood morbidities. Limited effects of iron can be expected regarding mental and motor development until the age of 2 years (we focused on the Bayley’s scales for mental and motor development due to more consistent reporting).

We conducted sub-group analyses stratified by type of intervention (fortification/ supplementation), intervention dose, a measure of baseline micronutrient status, as well as study quality. Sub-group analyses demonstrated that iron administered as fortificants significantly increased serum ferritin and hemoglobin levels, although to a lesser extent than supplements. This could be due to the presence of absorption inhibitors in fortified foods. For biofortification, the magnitude of the effect on iron and zinc status is unclear as to date no biofortification trials have been conducted with a focus on the 1000 days window. Yet, we would expect comparable effects as observed from fortification since both approaches deliver the micronutrients together with other dietary components. Sub-group analysis stratified by dose showed that daily iron doses as low as 6–8 mg increased serum ferritin levels. Larger effect sizes were observed for higher daily iron doses (8–10 mg), however, such doses might be difficult to attain for infants by dietary diversification or biofortification interventions alone. Surprisingly, baseline iron status did not explain much of the heterogeneity of the findings in our analysis. This is somewhat in contrast to previously published data [113,114] and could be ascribed to other factors influencing iron absorption and utilization in contexts of high exposure to inflammation [115]. But also, the categorization of studies into iron deficient versus iron sufficient population had to be done in a relatively crude manner.

We found that zinc supplementation at doses no higher than 10 mg/day increased the serum zinc concentrations of children and reduced the risk of suffering from zinc deficiency, whereas the effect of zinc administered in fortification trials, although in comparable concentrations as supplemental zinc, had no significant effect on serum zinc levels. We did not find any modification of the zinc intervention effects by child baseline zinc status. Sub-group analysis stratified by dose showed that daily zinc doses of 4–6 mg increased serum zinc by 0.9 µmol/L while larger effect sizes were observed for higher zinc doses 7–10 mg, which might be difficult to reach in children 6–23 months old through dietary means alone. Effects of zinc were significant for WHZ and WAZ, although the differences remained rather small, compared with controls. Zinc interventions probably do not have a measurable effect on the occurrence of respiratory infections and fever and effects on diarrhea are inconclusive.

Lastly, we found that, when zinc and iron were given together, their beneficial effects on serum zinc and serum iron levels were weaker than when each nutrient was given alone, suggesting that iron and zinc compete for absorption from the gut, a finding that has previously been posited [116].

Possible limitations of the review should be noted. First, many results exhibit large amounts of heterogeneity, which could not be explained by the factors we explored, and which are probably due to the differences in study design, the different types of interventions and reporting. Thus, the pooled estimates of some of the effects shown may be imprecise and results have to be interpreted with caution. Second, none of the studies meeting our inclusion criteria covered the full 1000 days window meaning that the impacts of the micronutrients could not be assessed holistically across the different life stages, but only in a compartmentalized manner. Thus, no statements can be made about whether a holistic approach covering the full 1000 days (iron and zinc interventions starting during pregnancy and continuing until 2 years of age) would have a synergistic or additive effect on certain outcome measures.

To illustrate this using ferritin levels and the Psychomotor Development Index, the absorption and utilization of dietary iron is affected by the subject’s iron status [117,118]. Thus, if an iron intervention given to pregnant women suffering from moderate to severe dietary iron deficiency anemia has a positive effect on the fetus’s iron status, infants born to such women would have a better iron status than control peers of the same age whose mothers received no iron [119]. Continuing the higher iron regime into infancy could actually decrease iron absorption/utilization by children in the intervention group, assuming they had benefited from exposure to prenatal use, while children in the prenatal control group who start receiving iron after birth may more efficiently absorb and utilize the little iron available. Taken together, it is possible that only small differences in iron status would be observed between the two groups at the age of two years. In contrast, with regard to the Psychomotor Development Index, one could imagine that a 1000-day intervention could lead to a synergistic effect, since some early neurodevelopmental constraints are partly irreversible [120]. Thus, a child exposed to prenatal iron would be primed for a better start early in life. Third, only few studies assessed the inflammatory status of the children and reported prevalence of inflammation and therefore it was not possible to stratify studies by level of inflammation. During inflammation the human body down regulates iron absorption, and thus studies with a high proportion of subjects suffering from inflammation would be expected to show less effects of iron on iron status. Also, in those populations, an effect of iron on hemoglobin and anemia prevalence could be masked by anemia of inflammation in the same subject.

To conclude, providing dietary or relatively low daily doses of iron and zinc to young children could be beneficial for their iron and zinc status, indicating that food based approaches can be useful tools to reduce the prevalence of iron and zinc deficiencies. However, it is questionable if an intervention would affect child outcomes throughout the whole 1000 days period, not least because of breast milk iron and zinc homeostasis [121,122,123,124]. More research assessing the impact of iron and zinc holistically over the different life stages (pregnancy, lactation, and early childhood) is required to understand the potential role of interventions provided throughout the 1000 days window.

## Figures and Tables

**Table 1:** Effects of prenatal iron interventions supplying ≤45 mg/day iron on birth weight and prevalence of low birth weight among offspring.

Variables	Mean Difference ^1^	Relative Risk	Studies, Participants (*n*)	*I*^2^ (%)	*p* Difference between Pooled Intervention and Control Groups
Birth outcomes					
Birthweight (g)	38 (−16; 91)		6, 13,627	58.2	0.17
Low birth weight (%)		0.69 (0.38; 1.26)	5, 12,845	63.1	0.23

^1^ 95% CI in parenthesis.

**Table 2:** Effects of daily iron administration (≤15 mg/day) to children 6–23 months on levels of hemoglobin, anemia, serum ferritin, iron deficiency, and iron deficiency anemia ^1^.

Variables	Mean Difference ^2^	Relative Risk	Studies, Participants (*n*)	*I*^2^ (%)	*p* Difference between Pooled Intervention and Control Groups (Bold Font) and Subgroups (Regular Font)
**Hb overall (g/dL)**	**4.1 (2.8; 5.3)**		**30, 6569**	**81.5**	**<0.001**
Iron dose					
<6 mg/day	−0.7 (−6.1; 4.7)		2, 220	73.1	0.12
6–8 mg/day	4.4 (2.1; 6.8)		7, 1864	83.9	
>8–10 mg/day	5.5 (3.4; 7.6)		13, 3068	83.8	
11–15 mg/day	2.7 (1.2; 4.2)		4, 403	80.7	
Type of intervention					
Supplementation	5.6 (3.4; 7.7)		15, 3516	86.4	<0.01
Fortification ^3^	2.6 (1.3; 3.9)		16, 3053	67.4	
RCT, quality rating					
highest	5.5 (3.3; 7.6)		12, 3403	87.1	<0.05
intermediate	3.2 (1.6; 4.8)		14, 2623	73.1	
lowest	1.3 (−2.9; 5.4)		4, 375	80.3	
**Anemia overall**		**0.59 (0.49; 0.70)**	**22, (5647)**	**73.8**	**<0.0001**
Iron dose					
6–8 mg/day		0.54 (0.44; 0.66)	7, 2089	18.4	0.32
>8–10 mg/day		0.59 (0.45; 0.77)	9, 2575	85.2	
11–15 mg/day		0.82 (0.51; 1.30)	3, 489	31.0	
**Serum ferritin (µg/dL)**	**17.3 (13.5; 21.2)**		**21, (4291)**	**95.1**	**<0.0001**
Iron dose					
<6 mg/day	5.8 (−14.8; 26.3)		2, 222	90.9	<0.01
6–8 mg/day	12.1 (2.6; 21.7)		5, 1261	96.4	
>8–10 mg/day	27.5 (16.0; 39.0)		9, 2068	96.4	
Type of intervention					
Supplementation	27.2 (18.2; 36.3)		8, 1747	90.1	<0.001
Fortification	11.3 (13.7; 21.4)		13, 2544	95.2	
RCT, quality rating					
highest	22.8 (15.2; 30.4)		9, 2351	93.0	0.08
intermediate	11.4 (6.6; 16.1)		9, 1619	93.0	
lowest	15.0 (7.0; 23.0)		3, 321	92.0	
Baseline ID prevalence					
Low (<15%)	27.0 (13.6; 40.4)		4, 276	60.5	0.76
High (≥15%)	32.4 (8.9; 55.9)		4, 1226	98.3	
Baseline mean serum ferritin ^4^					
Low (<29.2 µg/L)	18.5 (11.7; 25.3)		9, 1352	76.4	0.99
High (≥29.2 µg/L)	21.2 (11.5; 30.9)		8, 1698	94.0	
**ID overall**		**0.22 (0.14; 0.35)**	**13, 3698**	**86.3**	**<0.0001**
**IDA overall**		**0.20 (0.11; 0.37)**	**8, 3464**	**64.2**	**<0.0001**

^1^ Hb, hemoglobin, ID, iron deficiency; IDA, iron deficiency anemia; RCT, randomized controlled trial; ^2^ 95% CI in parenthesis; ^3^ Includes micronutrient powders and crushable tablets (foodlets); ^4^ Low is defined as below 50th percentile of all reported serum ferritin means; high is equal or above 50th percentile.

**Table 3:** Effects of daily iron administration (≤15 mg/day) to children 6–23 months on growth and mental and development outcomes ^1^.

Variables	Mean Difference ^2^	Relative Risk	Studies, Participants (*n*)	*I*^2^ (%)	*p* Difference between Pooled Intervention and Control Groups
Growth					
WAZ	−0.01 (−0.08; 0.05)		10, 3511	12.5	0.69
WHZ	0.02 (−0.06; 0.09)		9, 3297	36.8	0.62
HAZ	−0.02 (−0.08; 0.04)		10, 3511	8.2	0.57
Stunting		1.09 (0.92; 1.29)	4, 2159	0	0.33
Wasting		1.11 (0.84; 1.47)	4, 1975	0	0.45
Mental and motor development					
MDI	0.4 (−0.9; 1.7)		4, 1062	19.9	0.60
PDI	0.6 (−1.2; 2.4)		4, 1062	61.9	0.50

^1^ HAZ, height for age *z*-score; MDI, Bayley mental development index; PDI, Bayley psychomotor development index; WAZ, weight for age *z*-score; WHZ, weight for height *z*-score; ^2^ 95% CI in parenthesis.

**Table 4:** Effects of administering ≤21 mg/day zinc to pregnant women on birth weights and prevalence of low birth weight among their offspring.

Variables	Mean Difference ^1^	Relative Risk	Studies, Participants (*n*)	*I*^2^ (%)	*p* Difference between Pooled Intervention and Control Groups
Birth outcomes					
Birthweight (g)	1 (−32; 35)		8, 3457	0	0.94
Low birth weight		0.96 (0.67; 1.37)	6, 2518	0	0.83

^1^ 95% CI in parenthesis.

**Table 5:** Effects of daily zinc administration (≤10 mg) to children 6–23 month on serum zinc and prevalence of zinc deficiency in children ^1^.

Variables	Mean Difference ^2^	Relative Risk	Studies, Participants (*n*)	*I*^2^ (%)	*p* Difference between Pooled Intervention and Control Groups (Bold Font) and Subgroups (Regular Font)
**Serum zinc overall** (µmol/L)	2.0 (1.2; 2.9)		23, 8848	96.1	<0.0001
Zinc dose					
<4 mg/day	0.81 (−0.07; 1.68)		1, 256	55.5	0.05
4–<7 mg/day	0.9 (0.08; 1.71)		7, 1296	92.4	
7–10 mg/day	3.0 (1.5; 4.5)		14, 6867	98.5	
Type of intervention					
Supplementation	2.4 (1.5; 3.4)		19, 7732	98.5	<0.05
Fortification ^3^	0.3 (−0.1; 0.8)		6, 816	98.1	
Baseline ZD prevalence					
Low (<25%)	2.9 (0.2; 5.7)		4, 1231	97.8	0.15
High (≥25%)	2.8 (1.7; 3.9)		4, 2372	95.5	
Baseline mean serum zinc ^4^					
Low (<10.75 µg/L)	2.4 (0.7; 4.2)		7, 5635	98.7	0.96
High (≥10.75 µg/L)	2.3 (0.7; 3.9)		9, 2200	96.9	
**ZD overall**		0.47 (0.32; 0.69)	12, 6666	92.2	<0.001

^1^ RCT, randomized controlled trial; ZD, zinc deficiency; ^2^ 95% CI in parenthesis; ^3^ Includes micronutrient powders and crushable tablets (foodlets); ^4^ Low is defined as below 50th percentile of all reported serum zinc means; high is equal or above 50th percentile.

**Table 6:** Effects of daily zinc administration (≤10 mg) to children 6–23 month on growth ^1^.

Variables	Mean Difference ^2^	Relative Risk	Studies, Participants (*n*)	*I*^2^ (%)	*p* Difference between Pooled Intervention and Control Groups
Growth					
WAZ	0.05 (0.00; 0.10)		21, 7440	39.4	0.04
WHZ	0.04 (0.00; 0.08)		16, 6875	22.3	0.04
HAZ	0.00 (−0.04; 0.03)		20, 7340	9.2	0.80
Stunting		0.97 (0.90; 1.04)	6, 5443	0	0.39
Wasting		0.98 (0.79; 1.21)	6, 5441	32.0	0.82
Underweight		0.99 (0.90; 1.09)	5, 4793	10.7	0.83

^1^ HAZ, height for age *z*-score; WAZ, weight for age *z*-score; WHZ, weight for height *z*-score; ^2^ 95% CI in parenthesis.

## Supplementary Materials

The following are available online at http://www.mdpi.com/2072-6643/8/12/773/s1.
