# Supplementary material for: The Effect of Low Dose Iron and Zinc Intake on Child Micronutrient Status and Development during the First 1000 Days of Life: A Systematic Review and Meta-Analysis
Source: Nutrients. 2016 Nov 30;8(12):773. doi: 10.3390/nu8120773 (PMC5188428; doi:10.3390/nu8120773)
Supplement: Supplementary file 1 [file nutrients-08-00773-s001.docx]

Supplementary Materials: The Effect of Low Dose Iron and Zinc Intake on Child Micronutrient Status and Development during the First 1000 Days of Life: A Systematic Review and Meta-Analysis

Nicolai Petry, Ibironke Olofin, Erick Boy, Moira Donahue Angel and Fabian Rohner


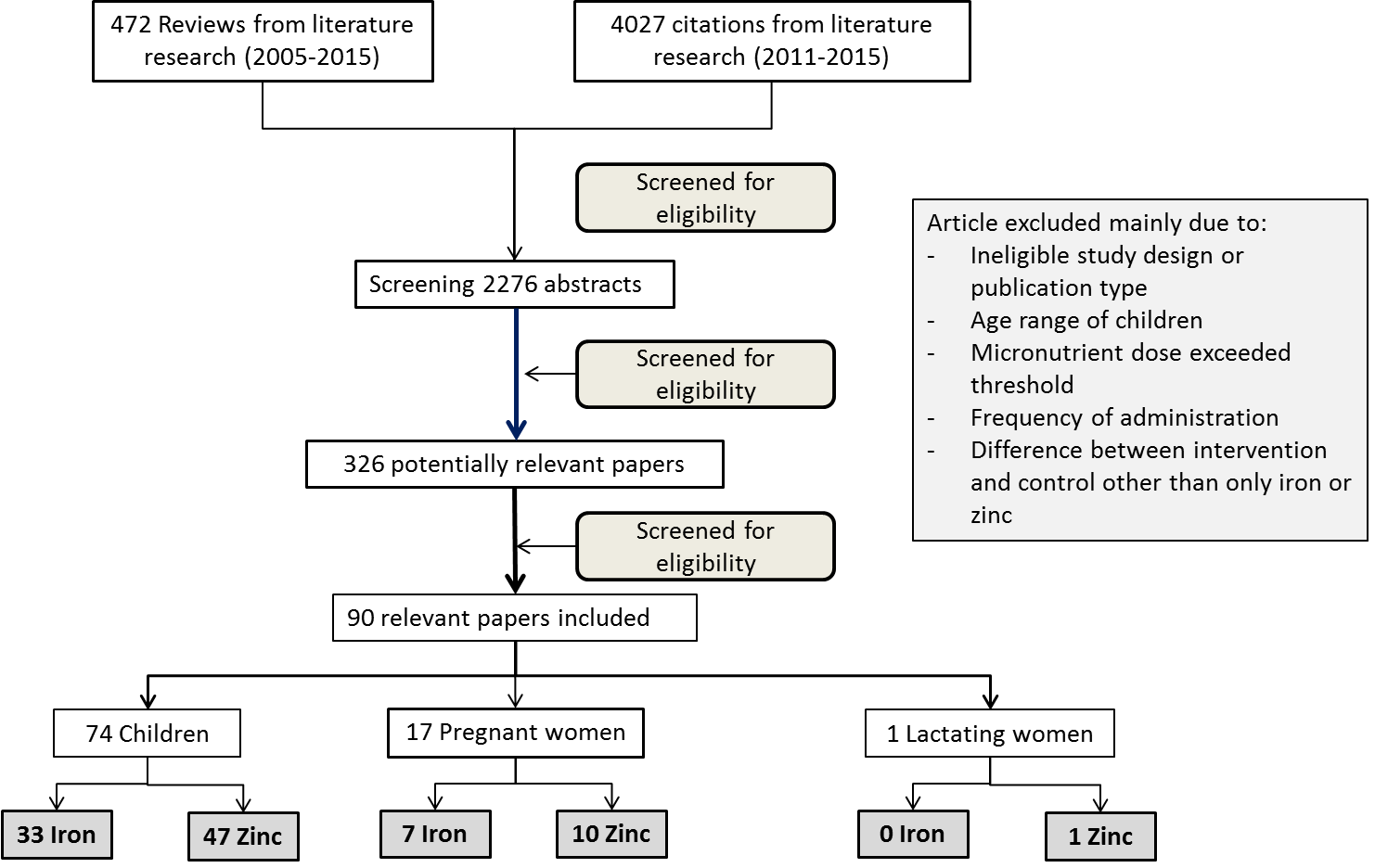


**Figure S1.** Summary of search strategy results.

Iron interventions:


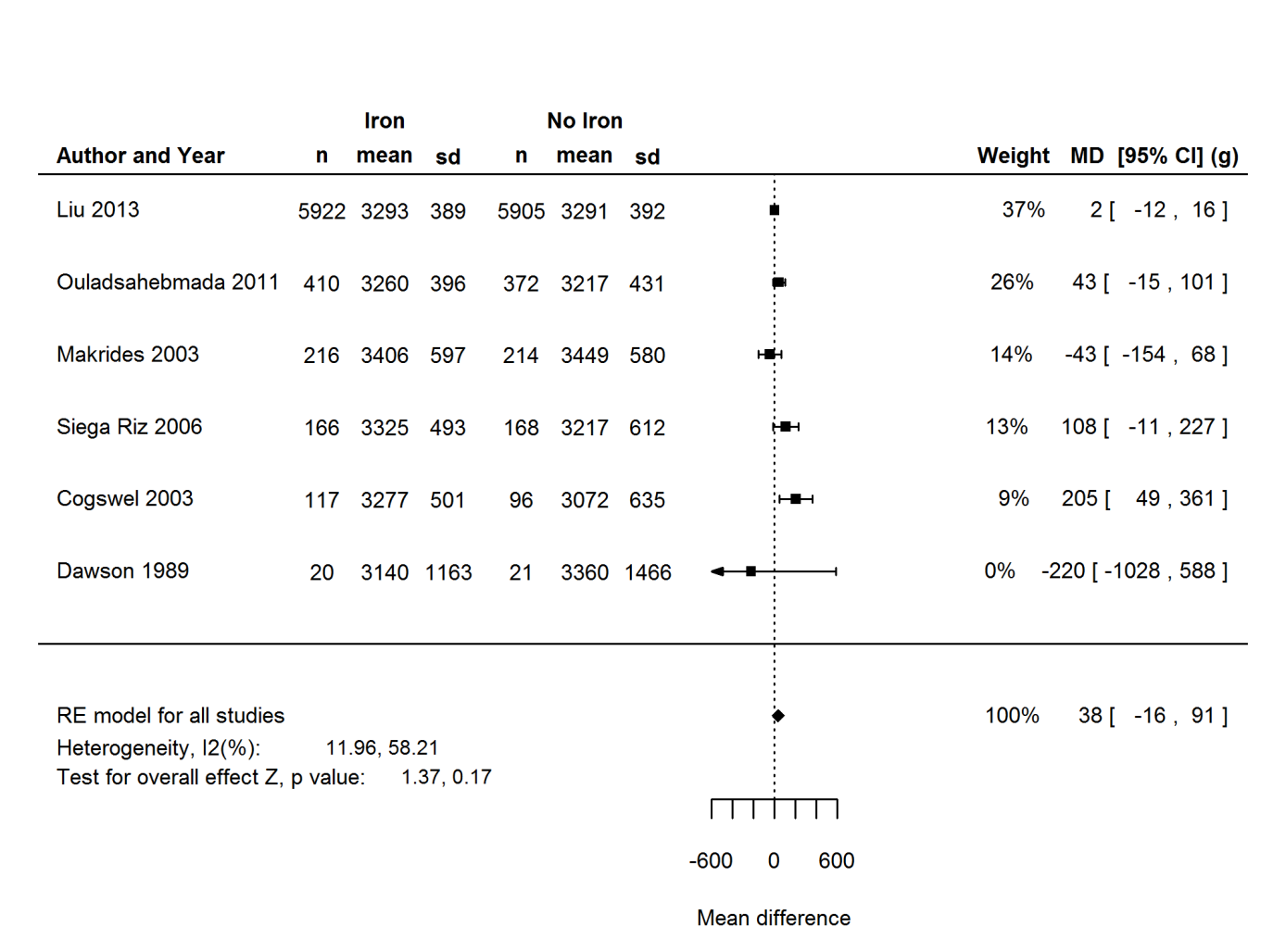


**Figure S2.** Forest plot summarizing the effect of interventions supplying up to 45 mg of additional iron daily to pregnant women on birth weight.


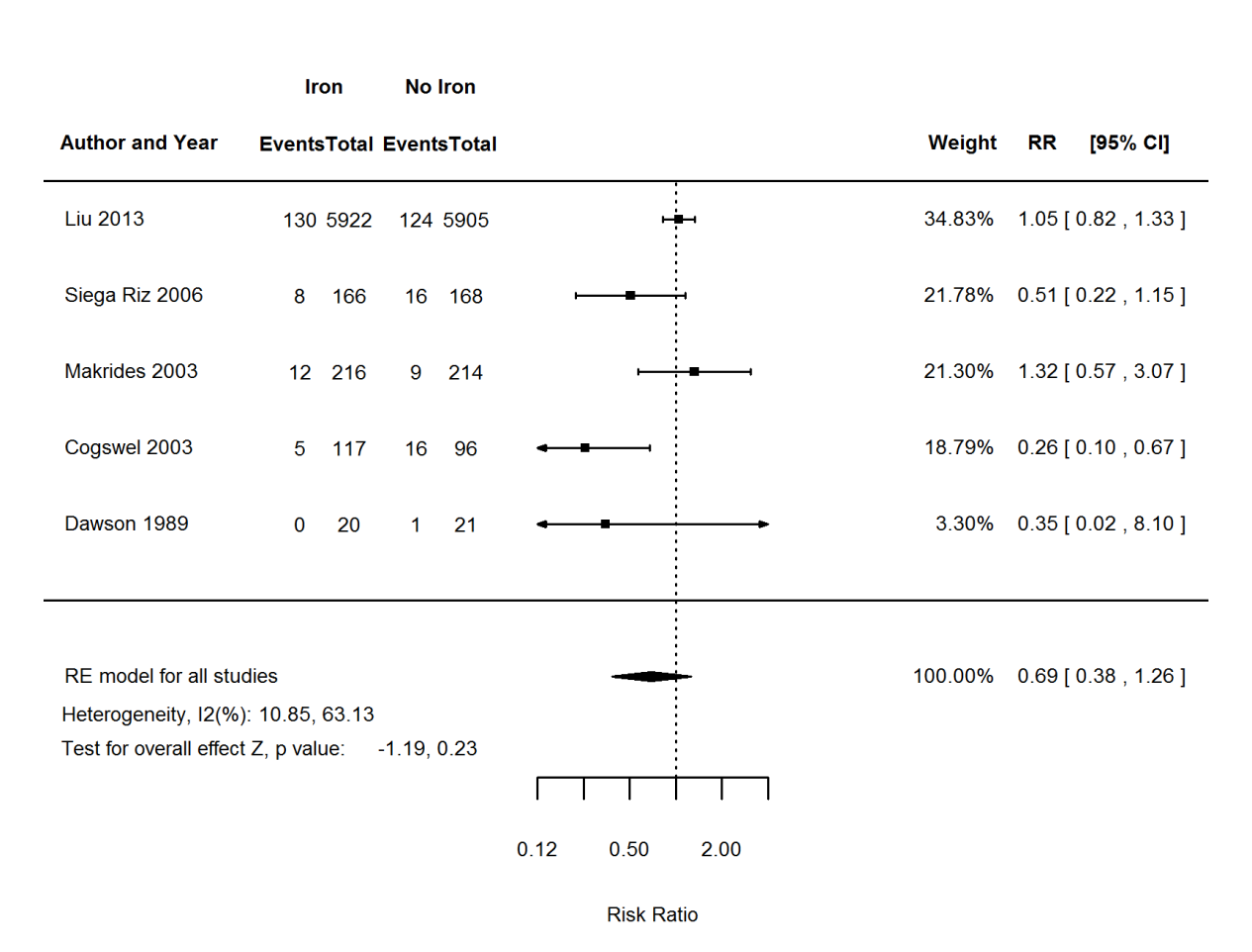


**Figure S3.** Forest plot summarizing the effect of interventions supplying up to 45 mg of additional iron daily to pregnant women on prevalence of low birth weight.


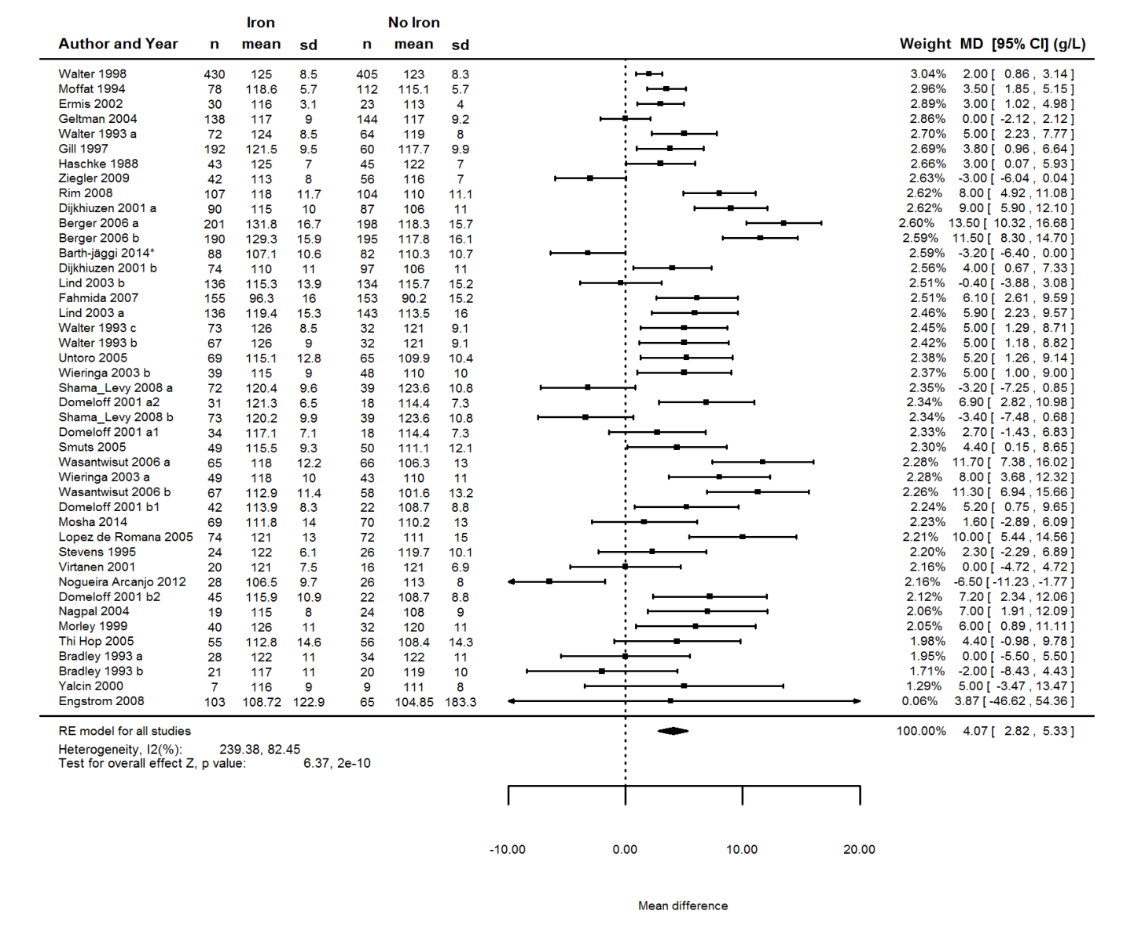


**Figure S4.** Forest plot summarizing the effect of interventions supplying up to 15 mg of additional iron daily to children 6 to 23 months old, on hemoglobin.


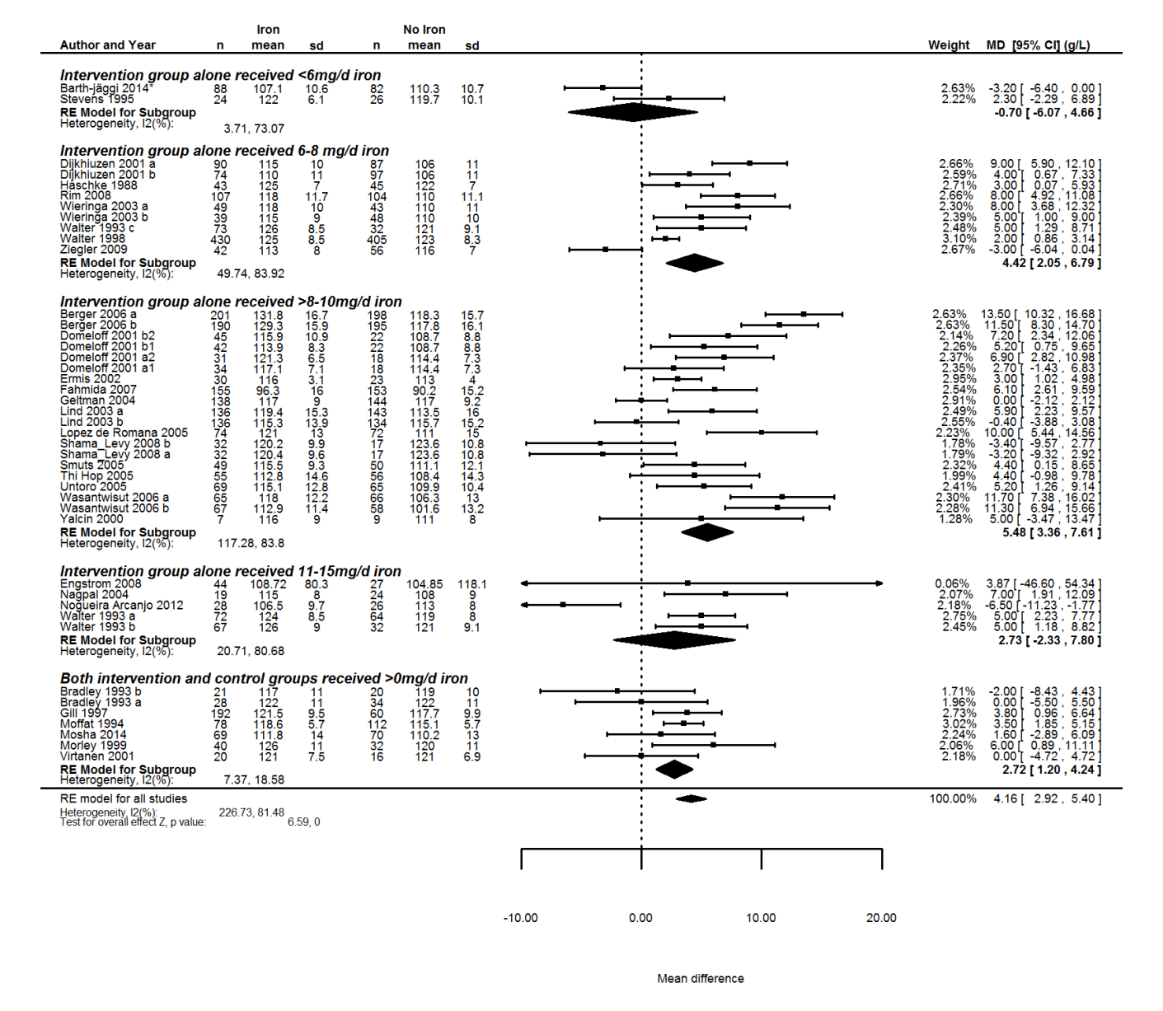


**Figure S5.** Forest plot summarizing the effect of interventions supplying up to 15 mg of additional iron daily to children 6 to 23 months old on hemoglobin; analysis stratified by dose.


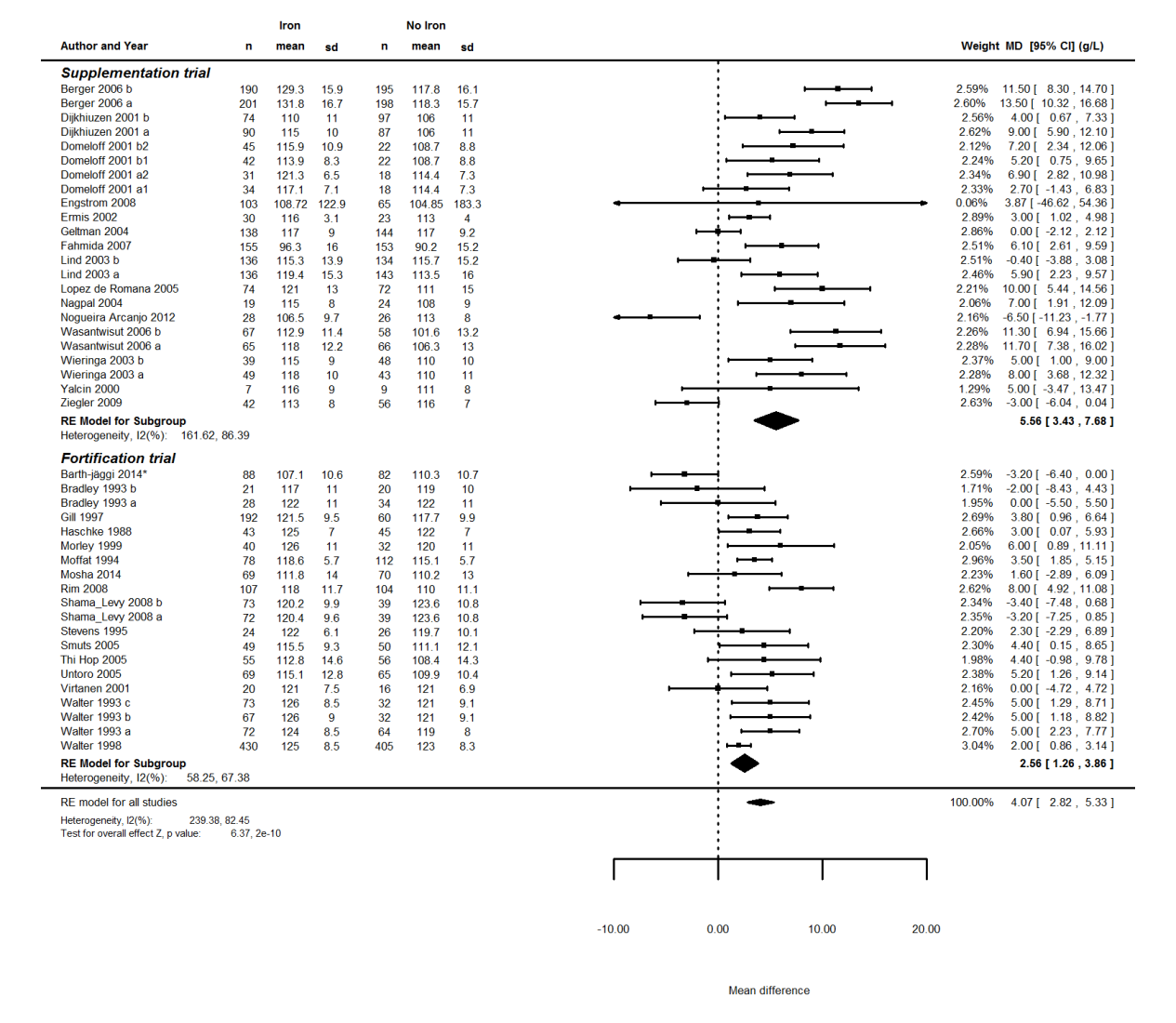


**Figure S6.** Forest plot summarizing the effect of interventions supplying up to 15 mg of additional iron daily on the hemoglobin levels of children 6 to 23 months old, stratified by intervention type.


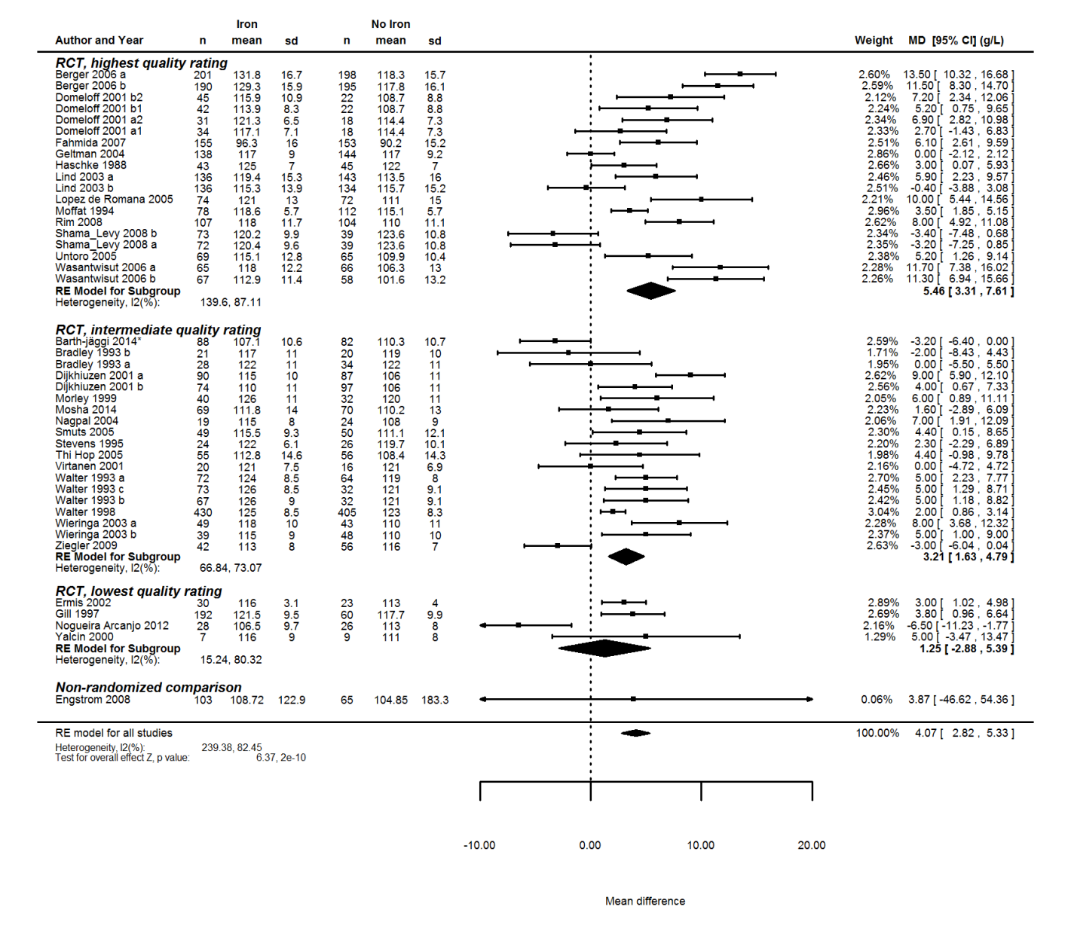


**Figure S7.** Forest plot summarizing the effect of interventions supplying up to 15 mg of additional iron daily on the hemoglobin concentrations of children 6 to 23 months old, stratified by quality rating.


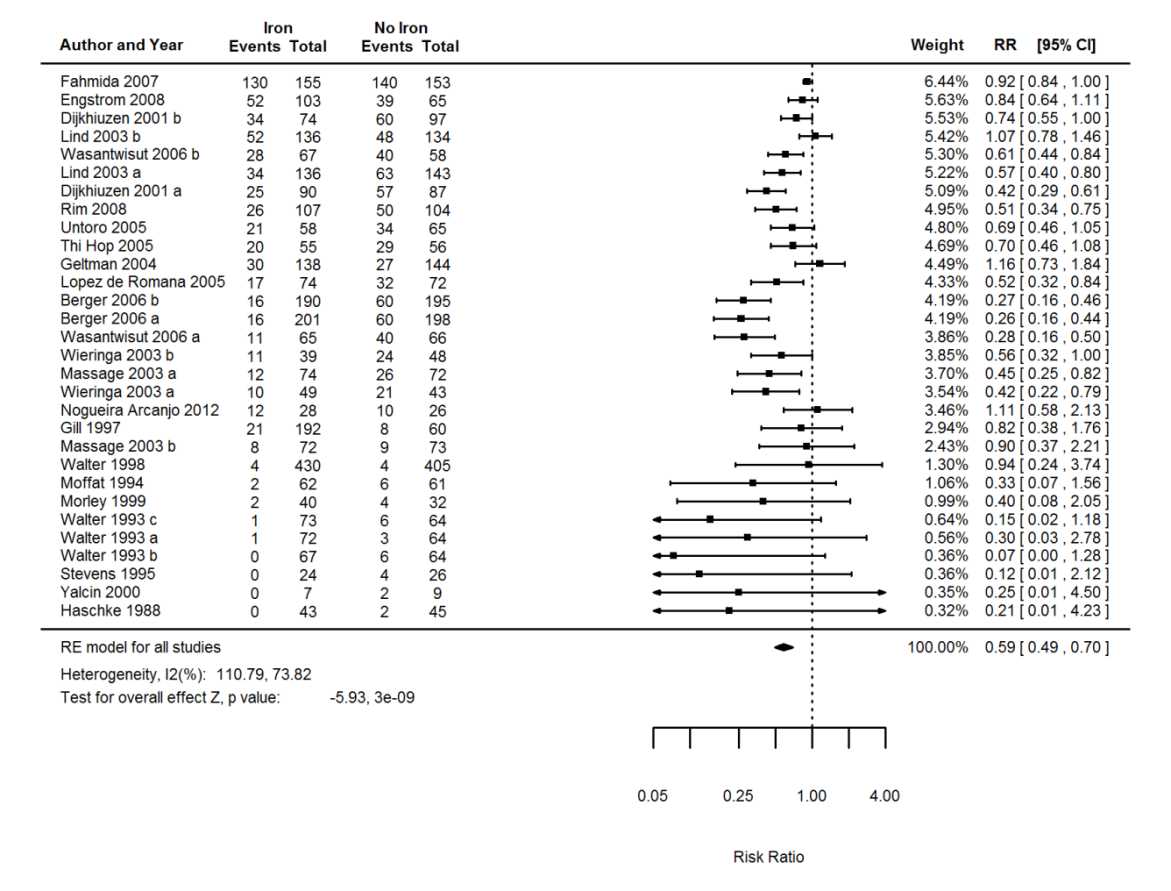


**Figure S8.** Forest plot summarizing the effect of interventions supplying up to 15 mg of additional iron daily to children 6 to 23 months old on the relative risk for anemia.


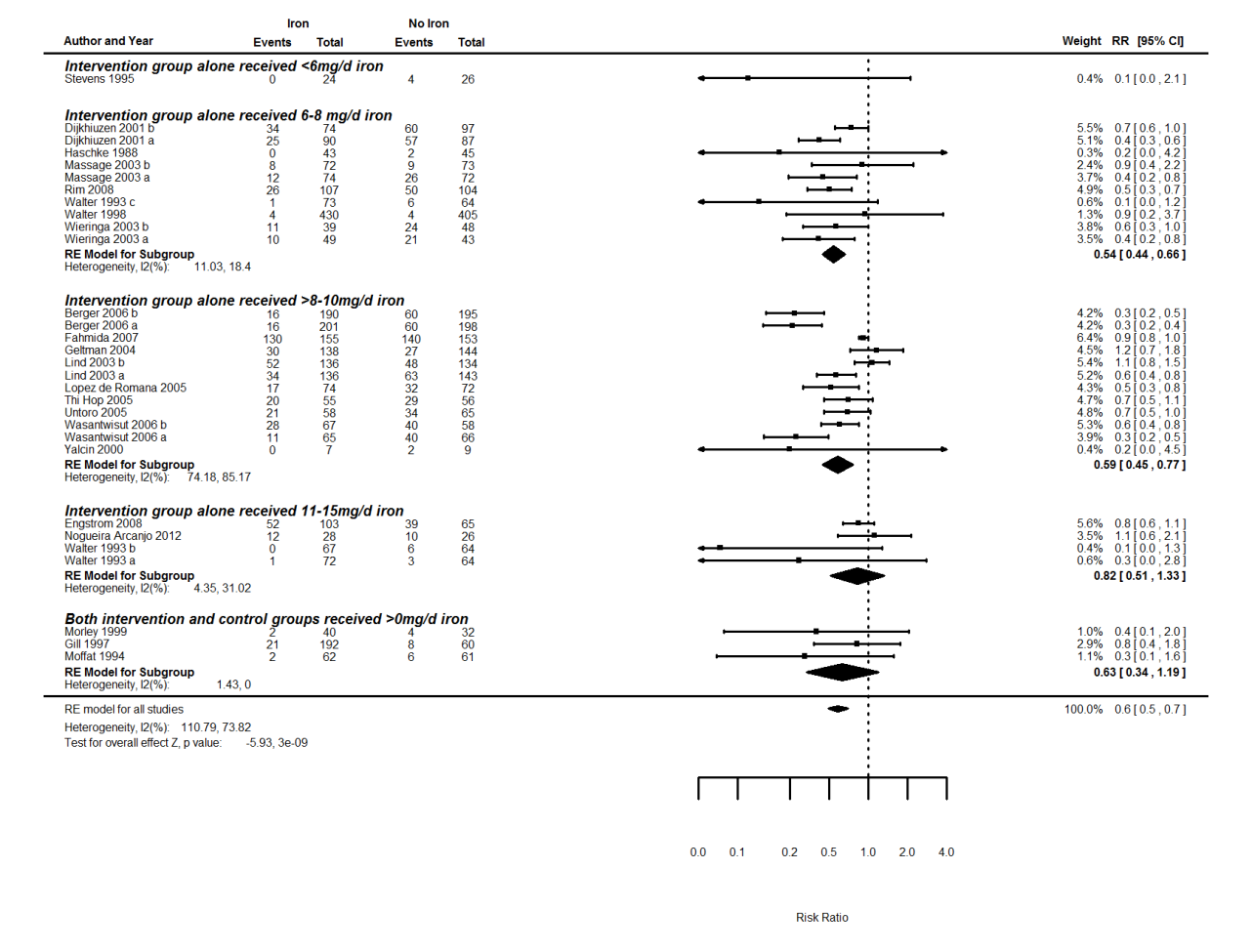


**Figure S9.** Forest plot summarizing the effect of interventions supplying up to 15 mg of additional iron daily to children 6 to 23 months old on the relative risk for anemia; analysis stratified by dose.


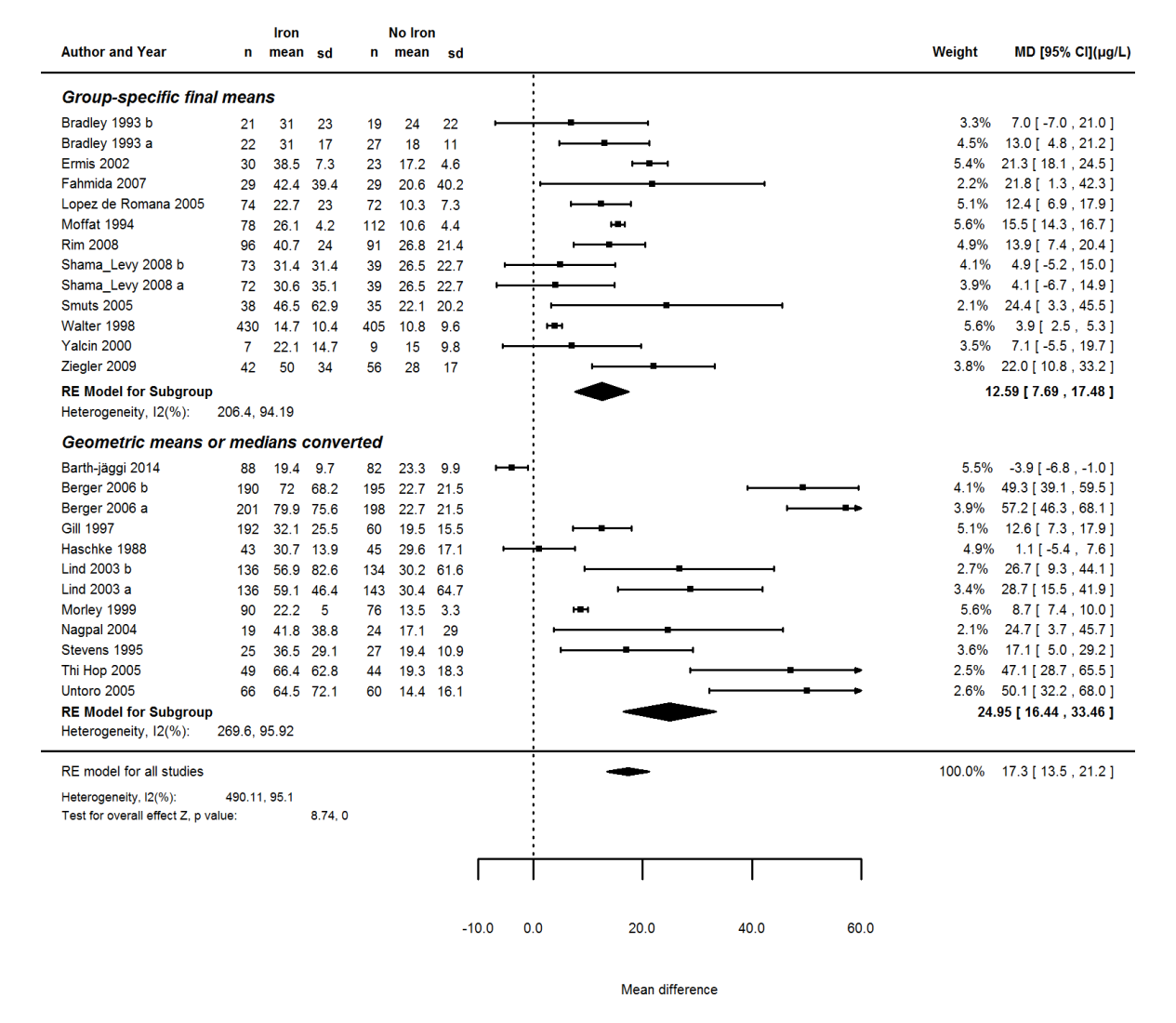


**Figure S10.** Forest plot summarizing the effect of interventions supplying up to 15 mg of additional iron daily to children 6 to 23 months old on serum ferritin.


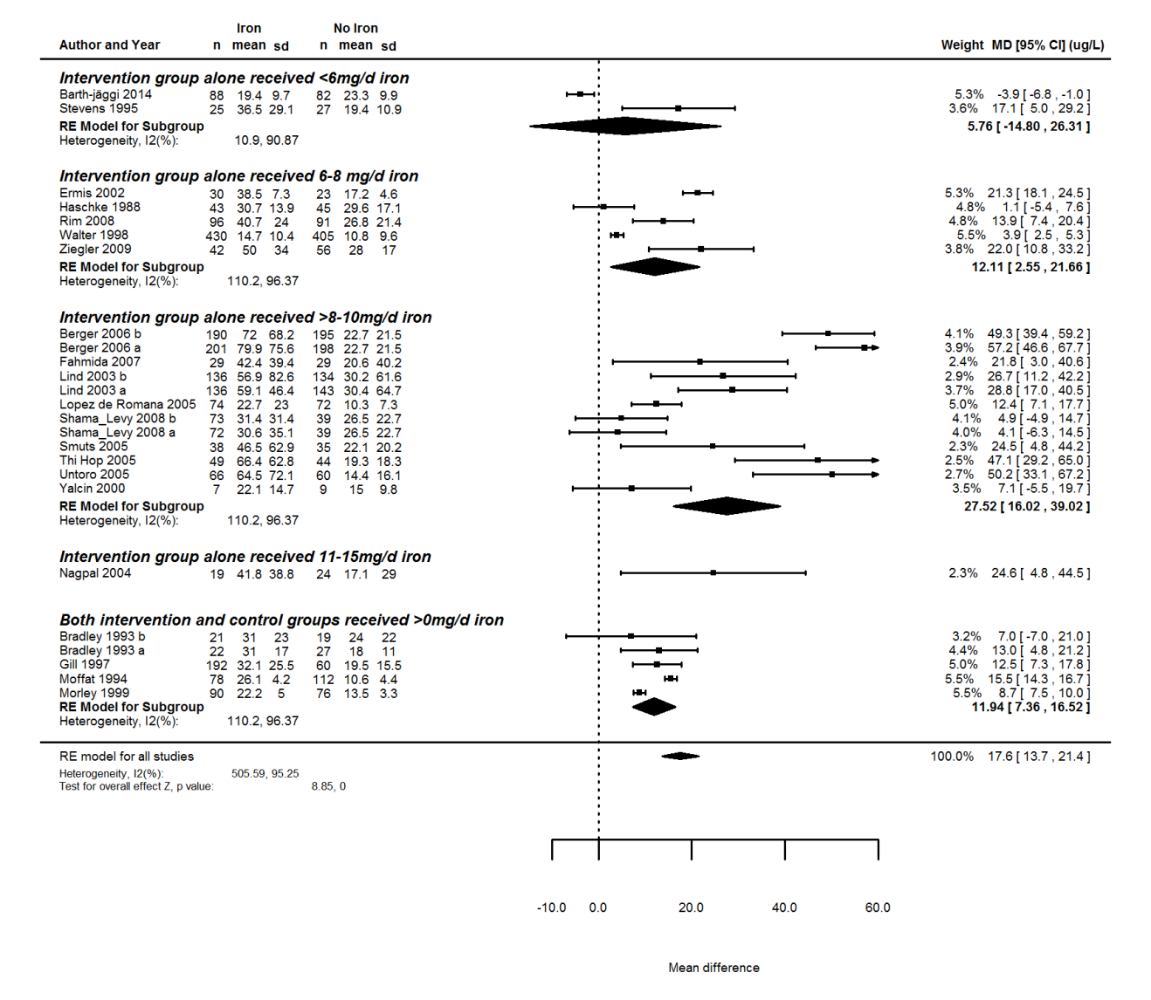


**Figure S11.** Forest plot summarizing the effect of interventions supplying up to 15 mg of additional iron daily to children 6 to 23 months old on serum ferritin; analysis stratified by dose.


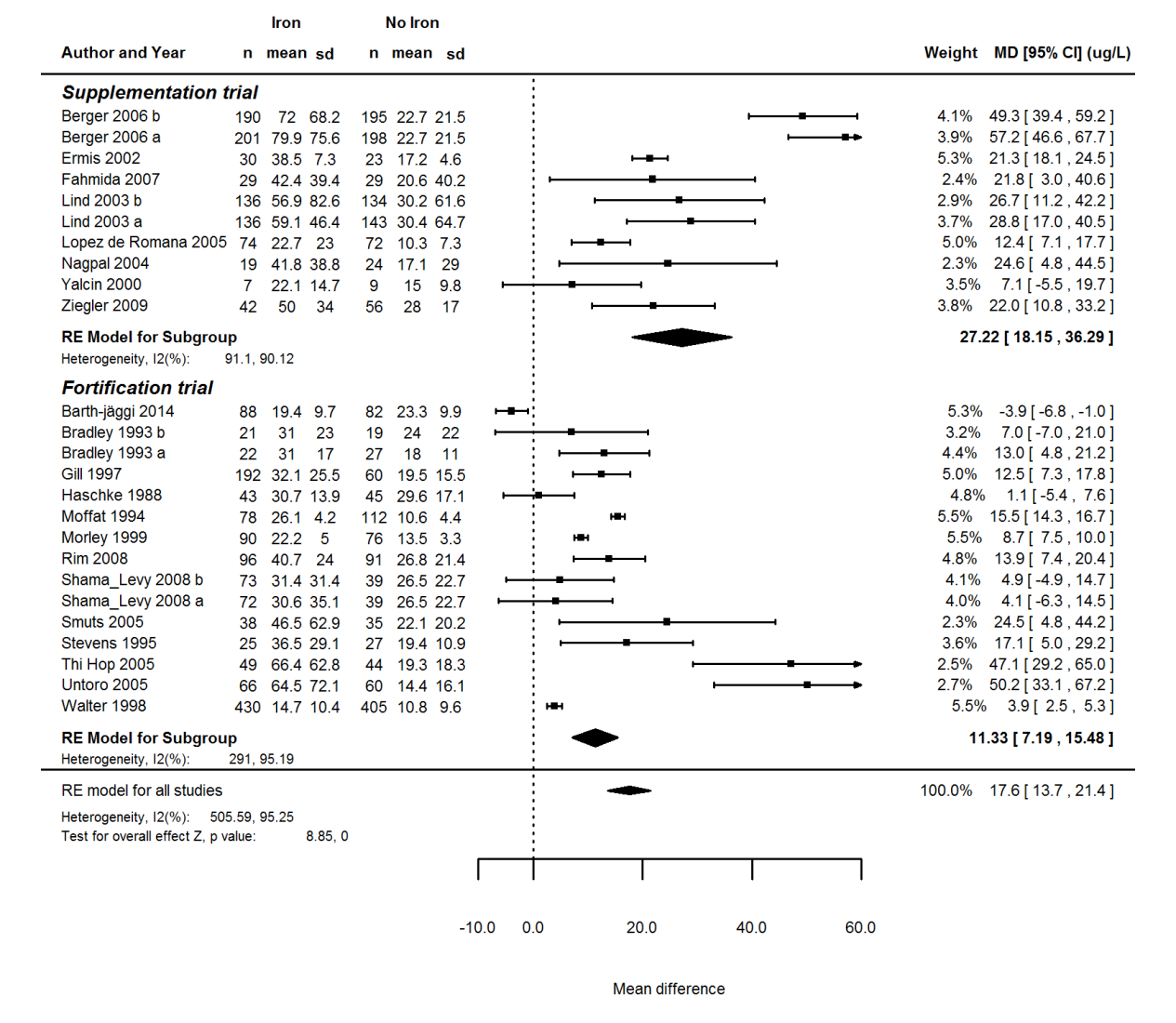


**Figure S12.** Forest plot summarizing the effect of interventions supplying up to 15 mg of additional iron daily to children 6 to 23 months old on serum ferritin; analysis stratified by type of intervention.


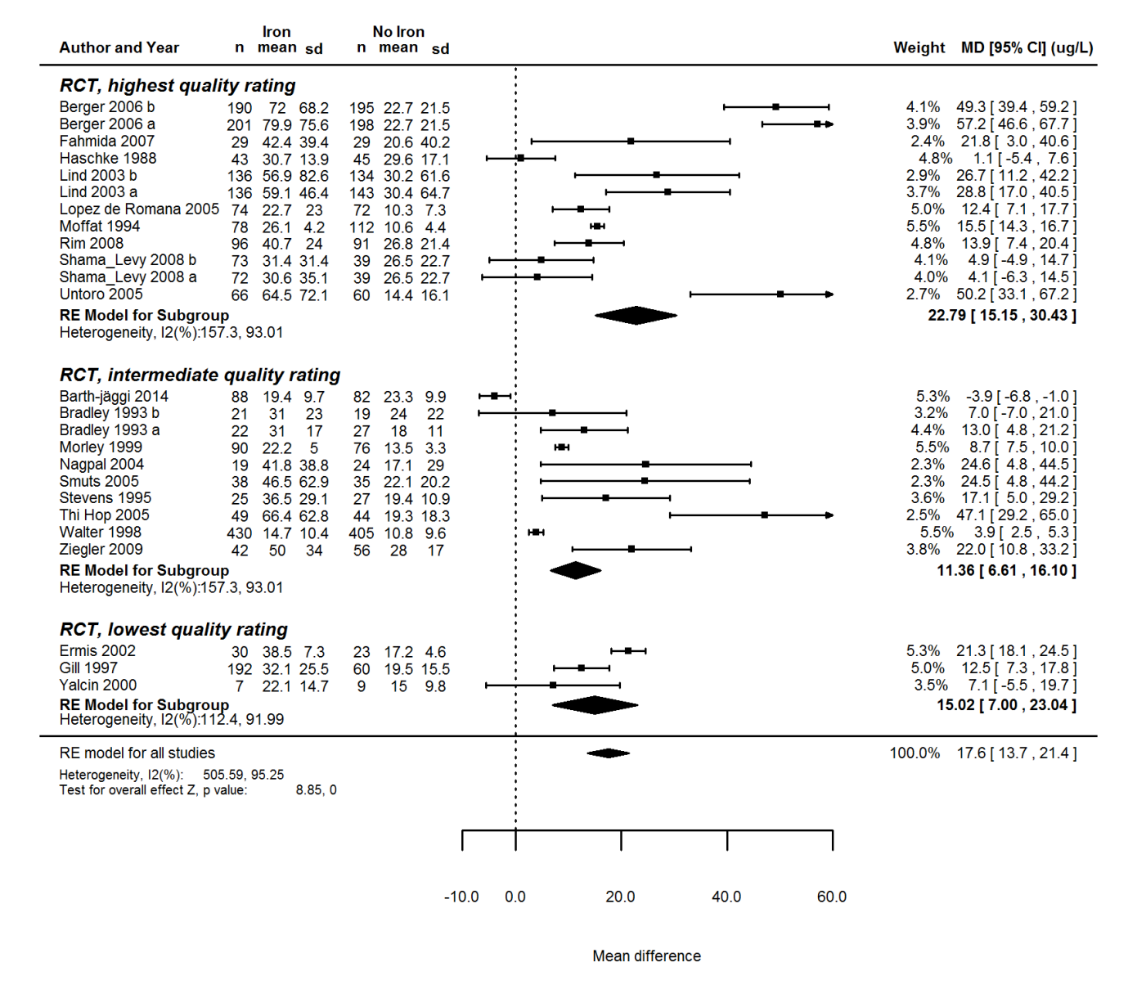


**Figure S13.** Forest plot summarizing the effect of interventions supplying up to 15 mg of additional iron daily on the serum ferritin concentrations of children 6 to 23 months old, stratified by quality rating.


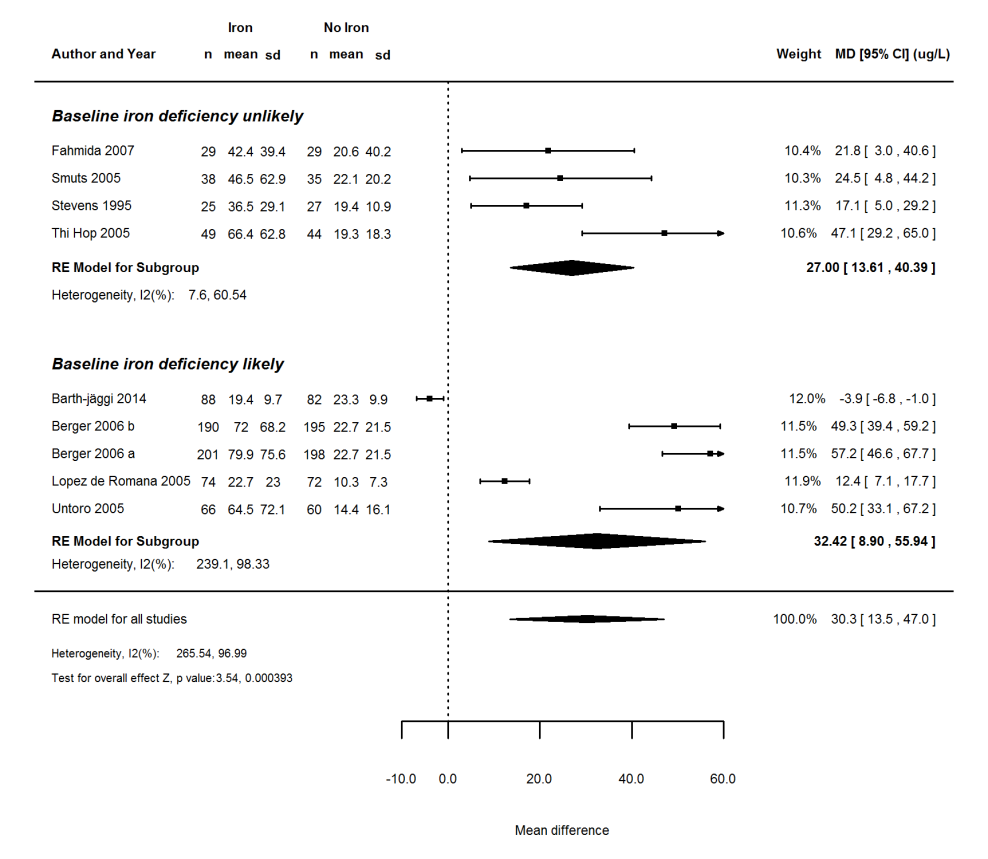


**Figure S14.** Forest plot summarizing the effect of interventions supplying up to 15 mg of additional iron daily to children 6 to 23 months old on ID; analysis stratified by baseline status.


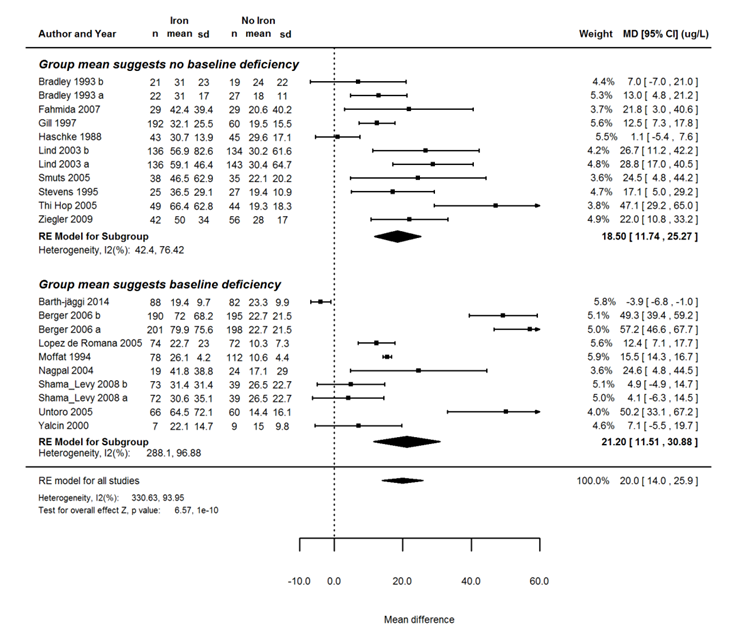


**Figure S15.** Forest plot summarizing the effect of interventions supplying up to 15 mg of additional iron daily to children 6 to 23 months old on serum ferritin; analysis stratified by baseline status.


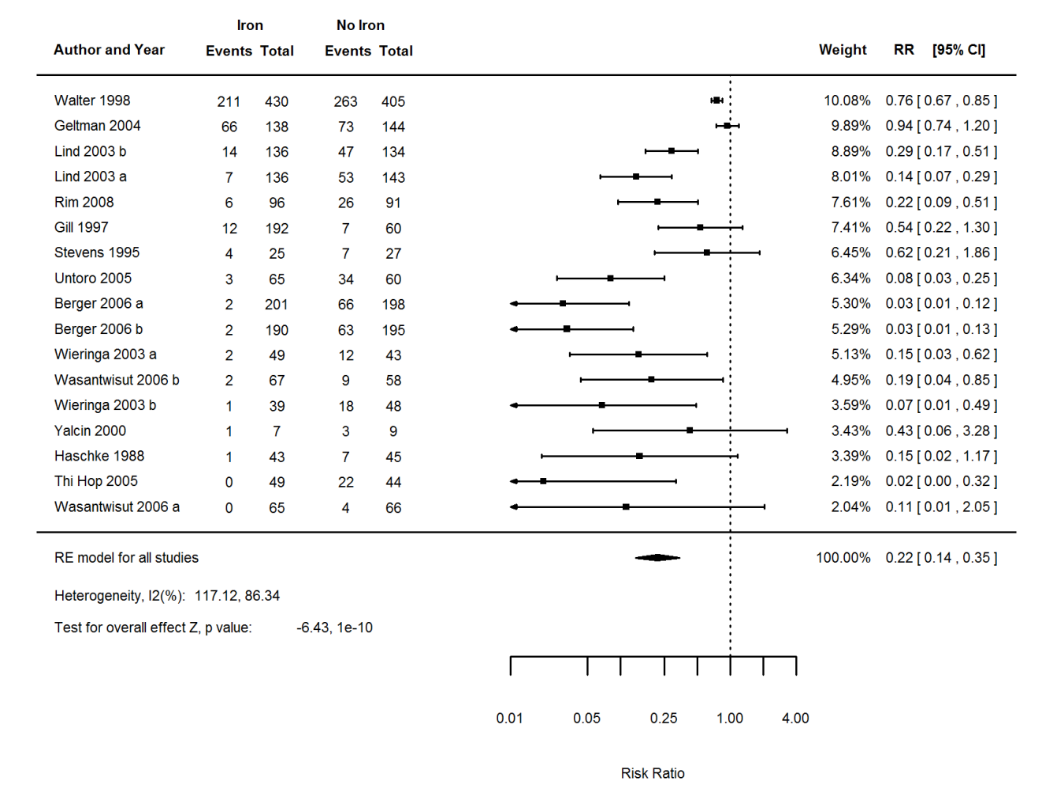


**Figure S16.** Forest plot summarizing the effect of interventions supplying up to 15 mg of additional iron daily to children 6 to 23 months old on iron deficiency.


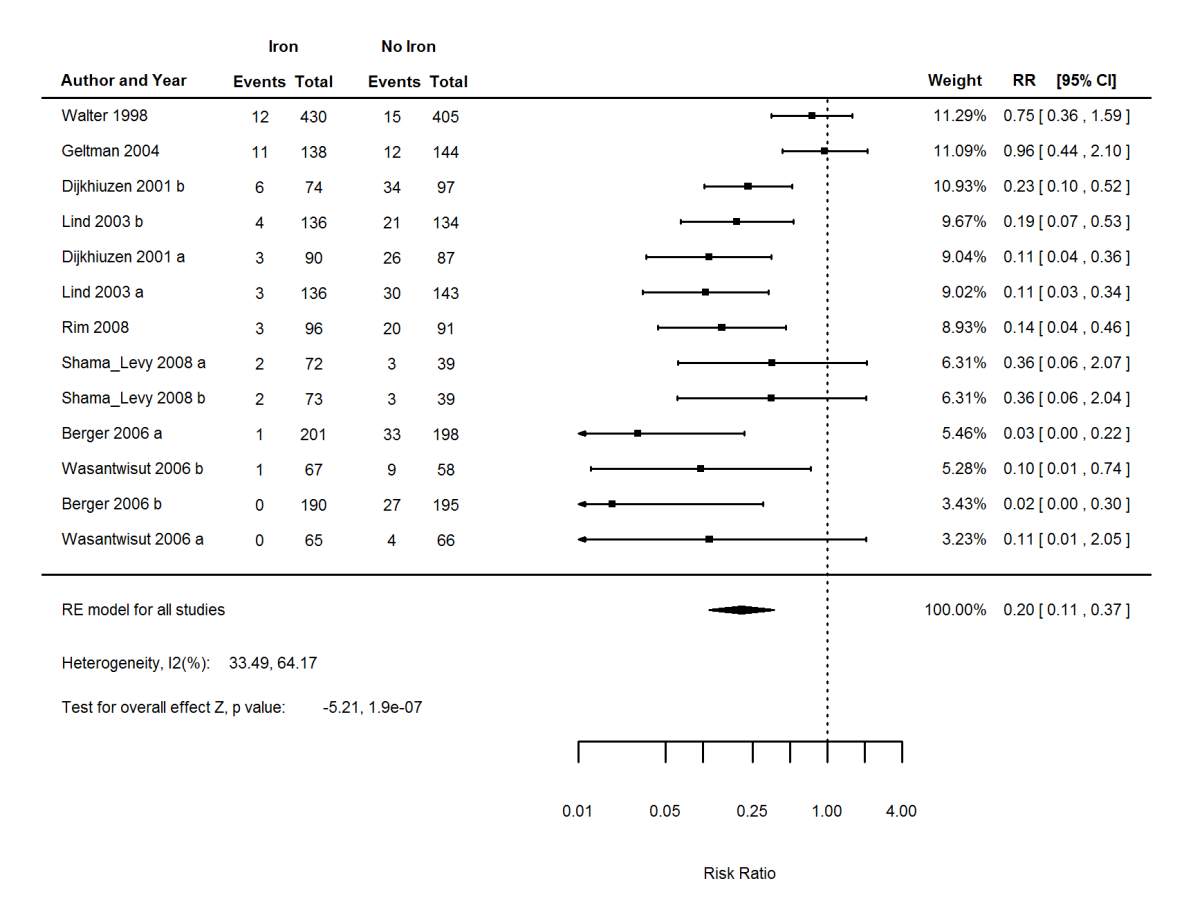


**Figure S17.** Forest plot summarizing the effect of interventions supplying up to 15 mg of additional iron daily to children 6 to 23 months old on iron deficiency anemia.


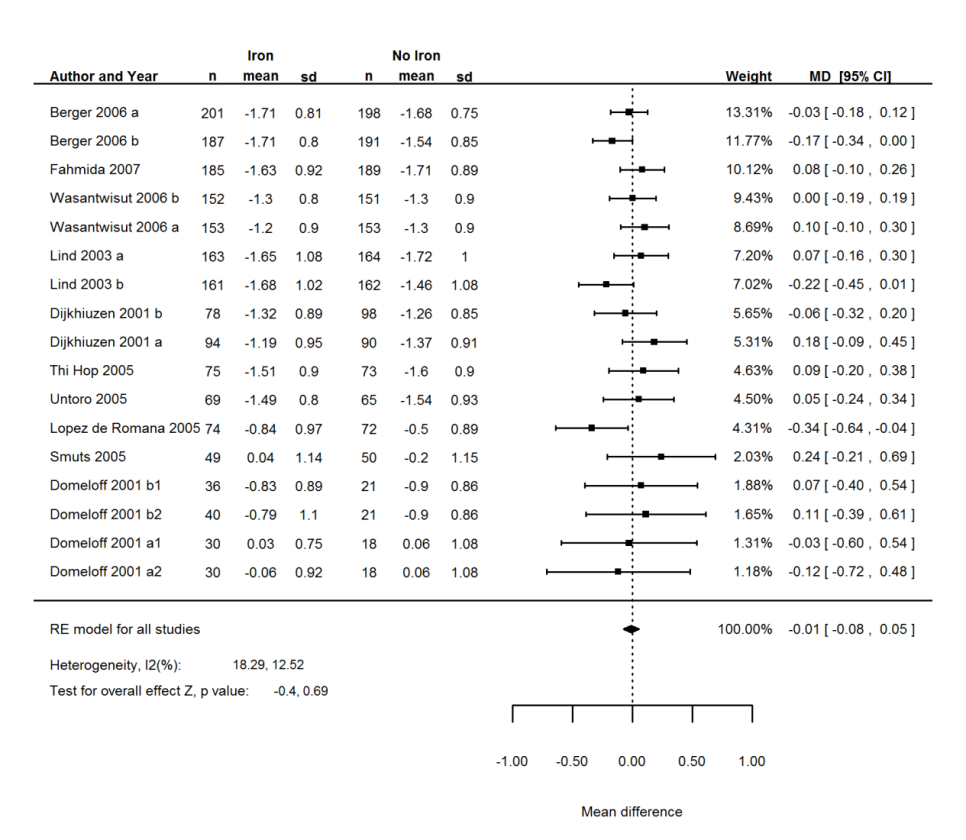


**Figure S18.** Forest plot summarizing the effect of interventions supplying up to 15 mg of additional iron daily to children 6 to 23 months old on weight-for-age *Z* scores (WAZ).


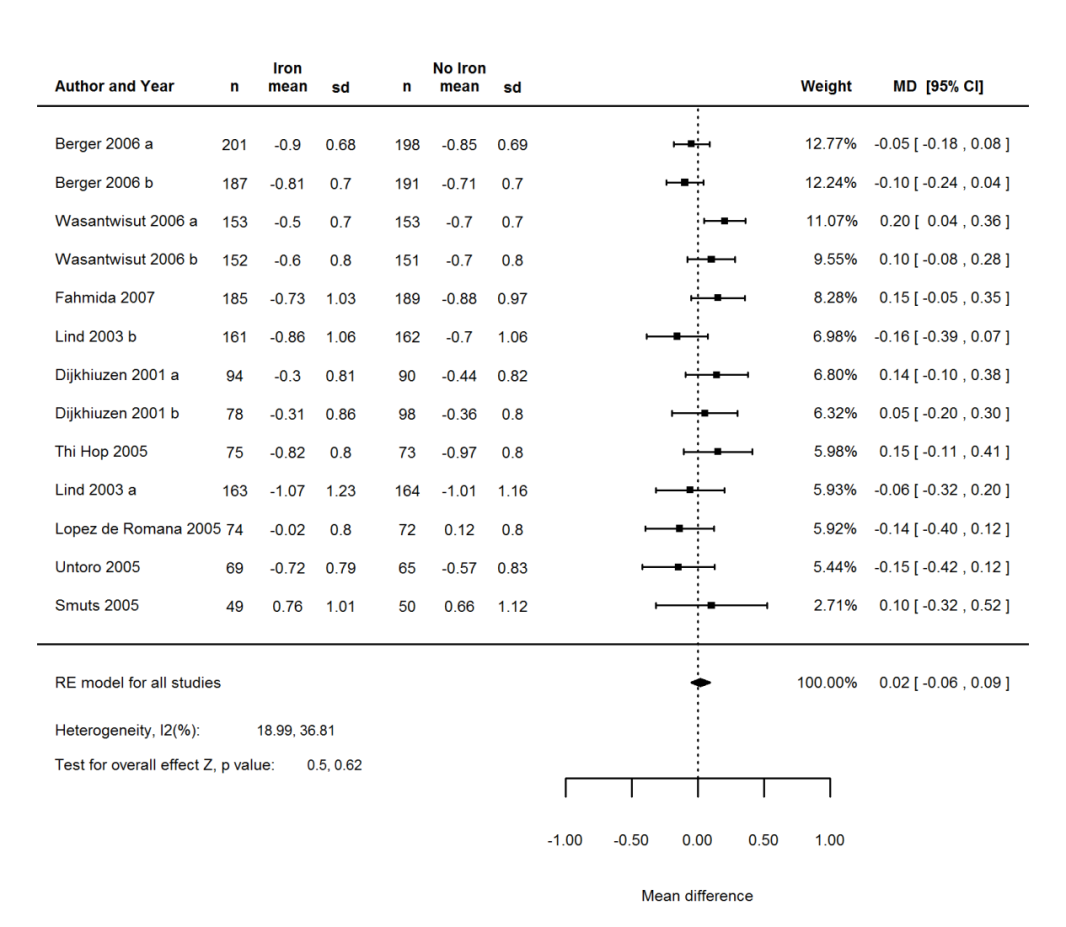


**Figure S19.** Forest plot summarizing the effect of interventions supplying up to 15 mg of additional iron daily to children 6 to 23 months old on weight-for-height *Z* scores (WHZ).


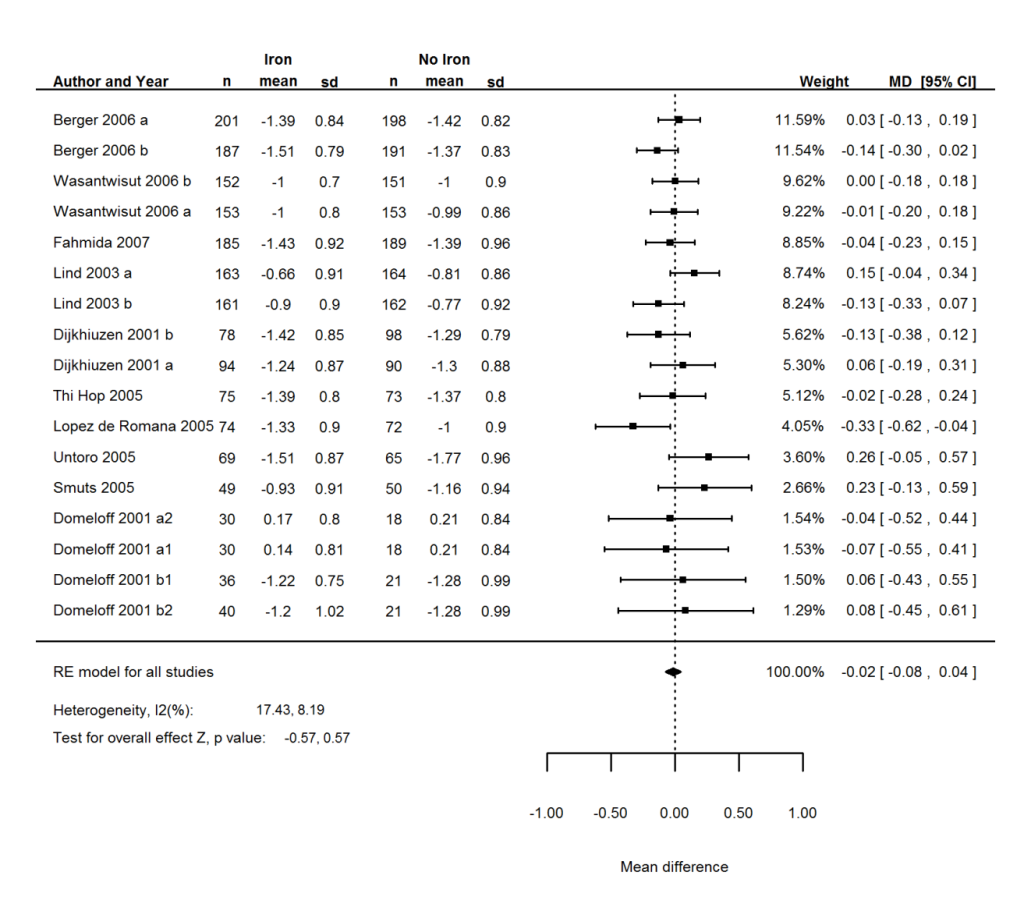


**Figure S20.** Forest plot summarizing the effect of interventions supplying up to 15 mg of additional iron daily to children 6 to 23 months old on height-for-age *Z* scores (HAZ).


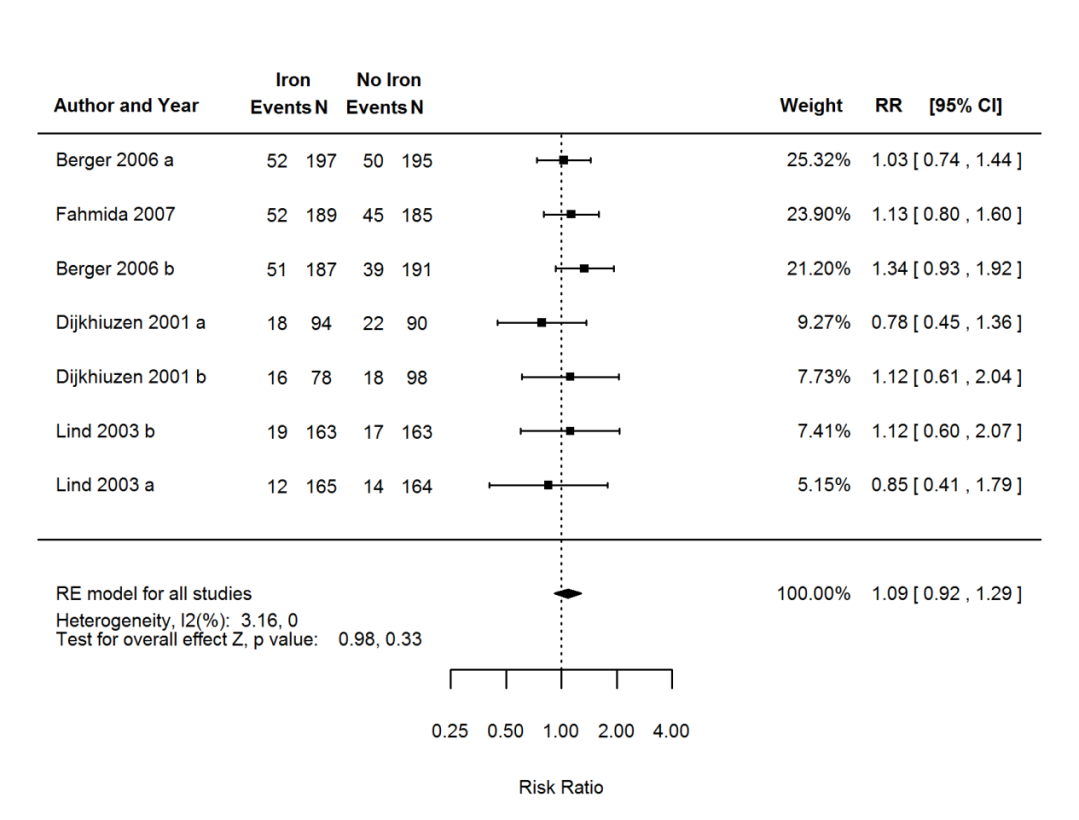


**Figure S21.** Forest plot summarizing the effect of interventions supplying up to 15 mg of additional iron daily to children 6 to 23 months old on the risk of childhood stunting.


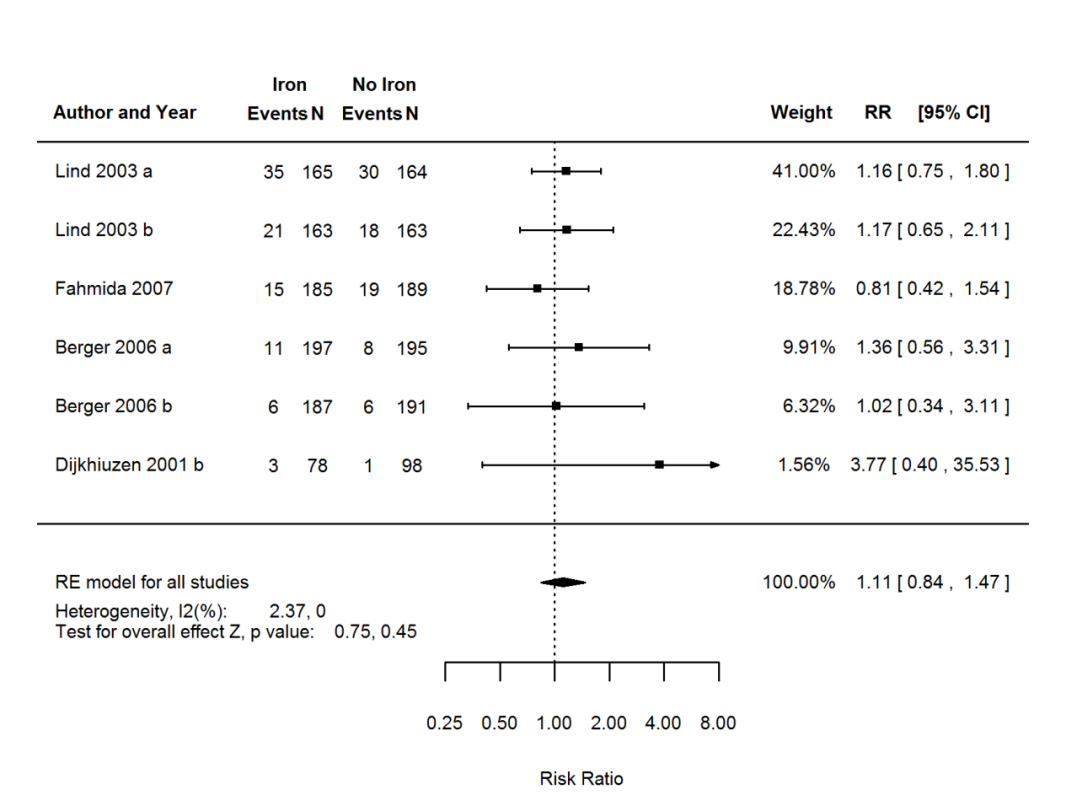


**Figure S22.** Forest plot summarizing the effect of interventions supplying up to 15 mg of additional iron daily to children 6 to 23 months old on the risk of childhood wasting.


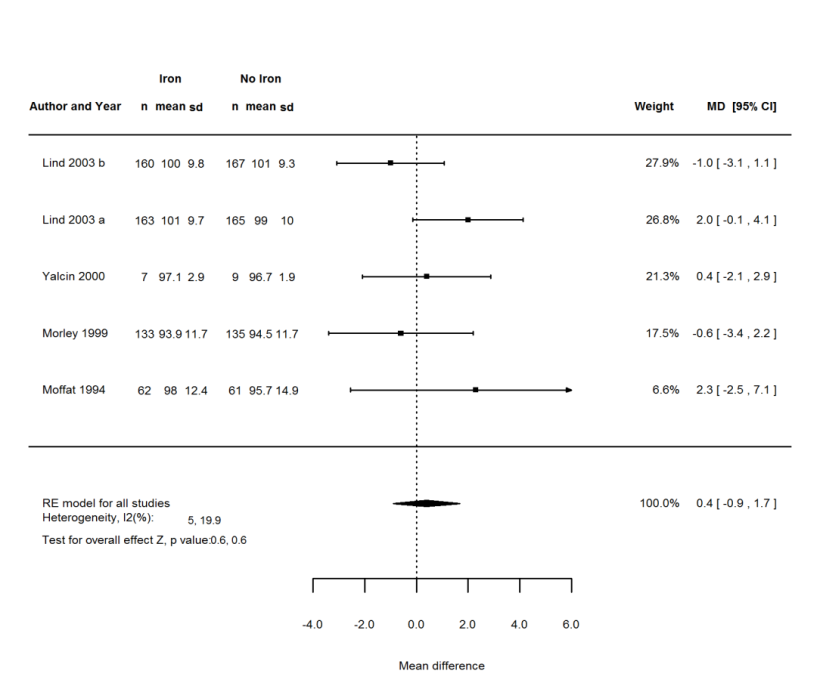


**Figure S23.** Forest plot summarizing the effect of interventions supplying up to 15 mg of additional iron daily to children 6 to 23 months old on the Bayley Mental Development Index (MDI).


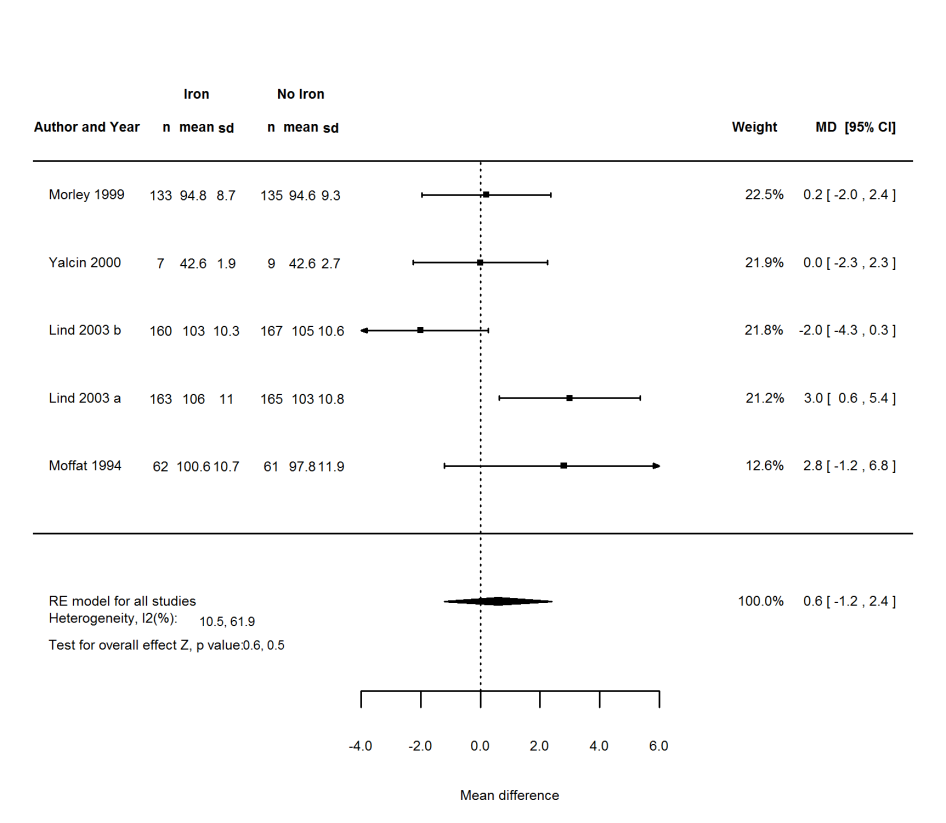


**Figure S24.** Forest plot summarizing the effect of interventions supplying up to 15 mg of additional iron daily to children 6 to 23 months old on the Bayley Psychomotor (PDI).

Zinc interventions:


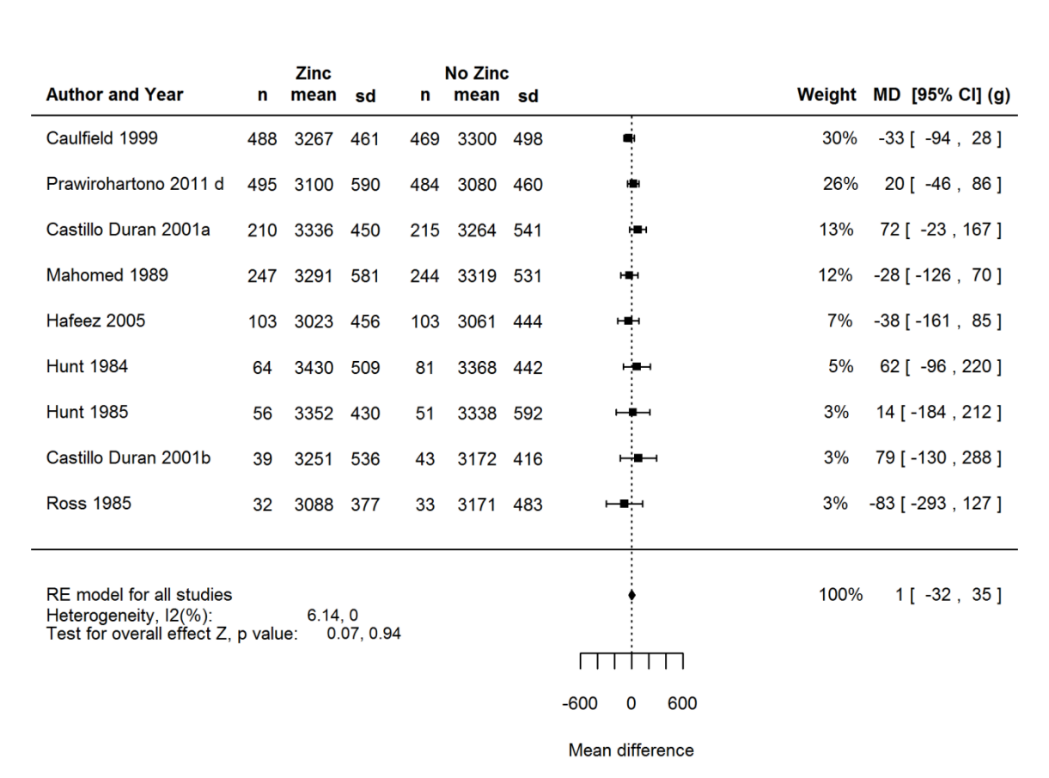


**Figure S25.** Forest plot summarizing the effect of interventions supplying up to 21 mg of additional zinc daily to pregnant women, on birth weight.


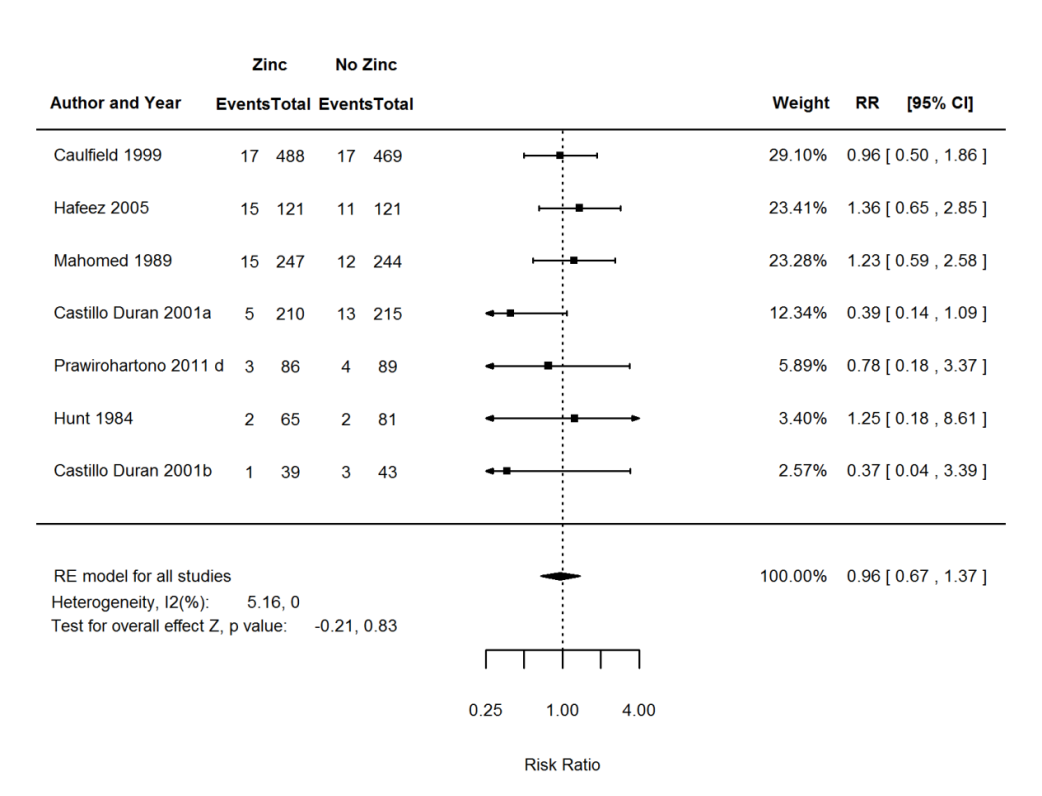


**Figure S26.** Forest plot summarizing the effect of interventions supplying up to 21 mg of additional zinc daily to pregnant women, on prevalence of low birth weight.


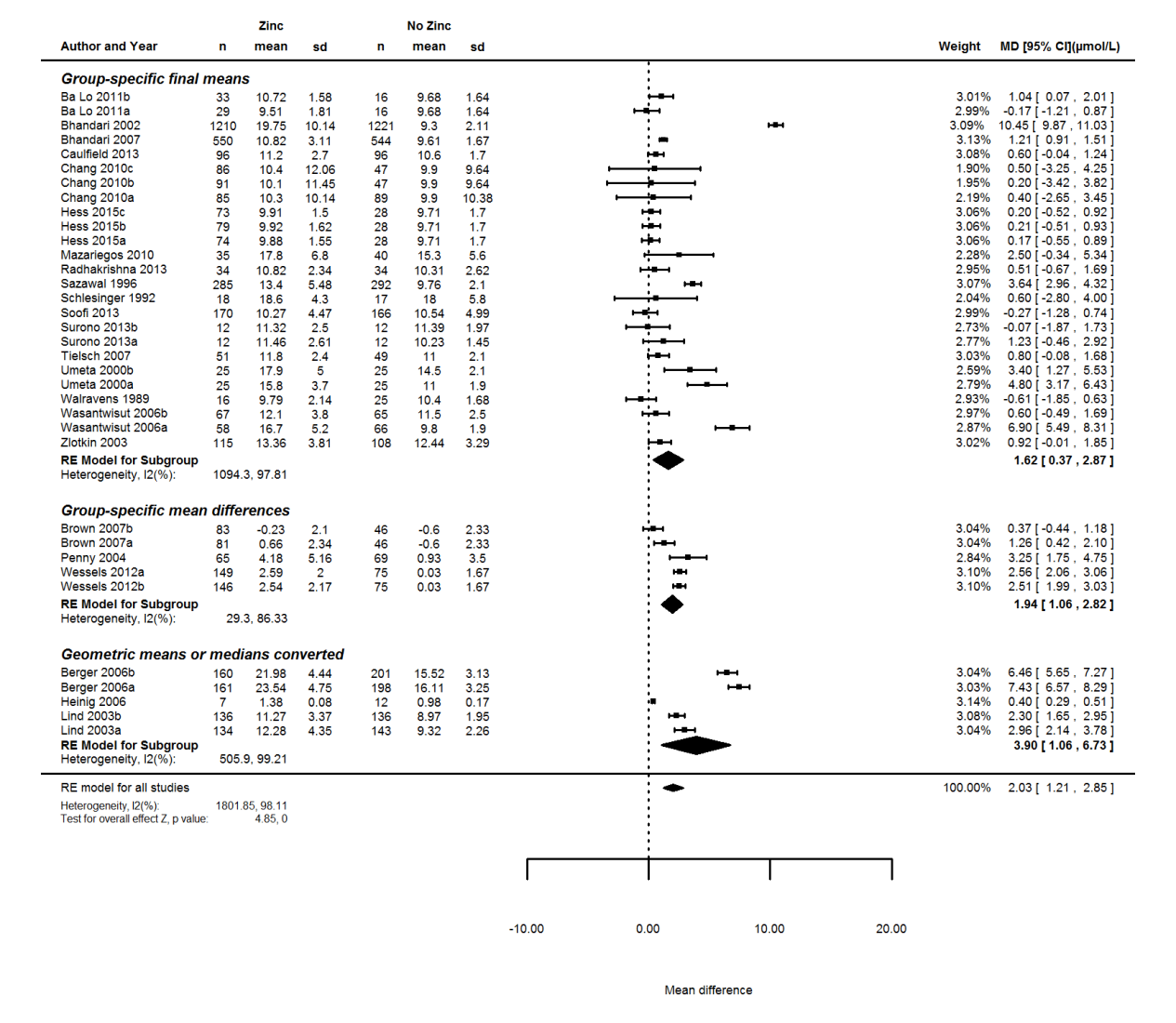


**Figure S27.** Forest plot summarizing the effect of interventions supplying up to 10 mg of additional zinc daily to children 6 to 23 months old, on serum or plasma zinc concentrations.


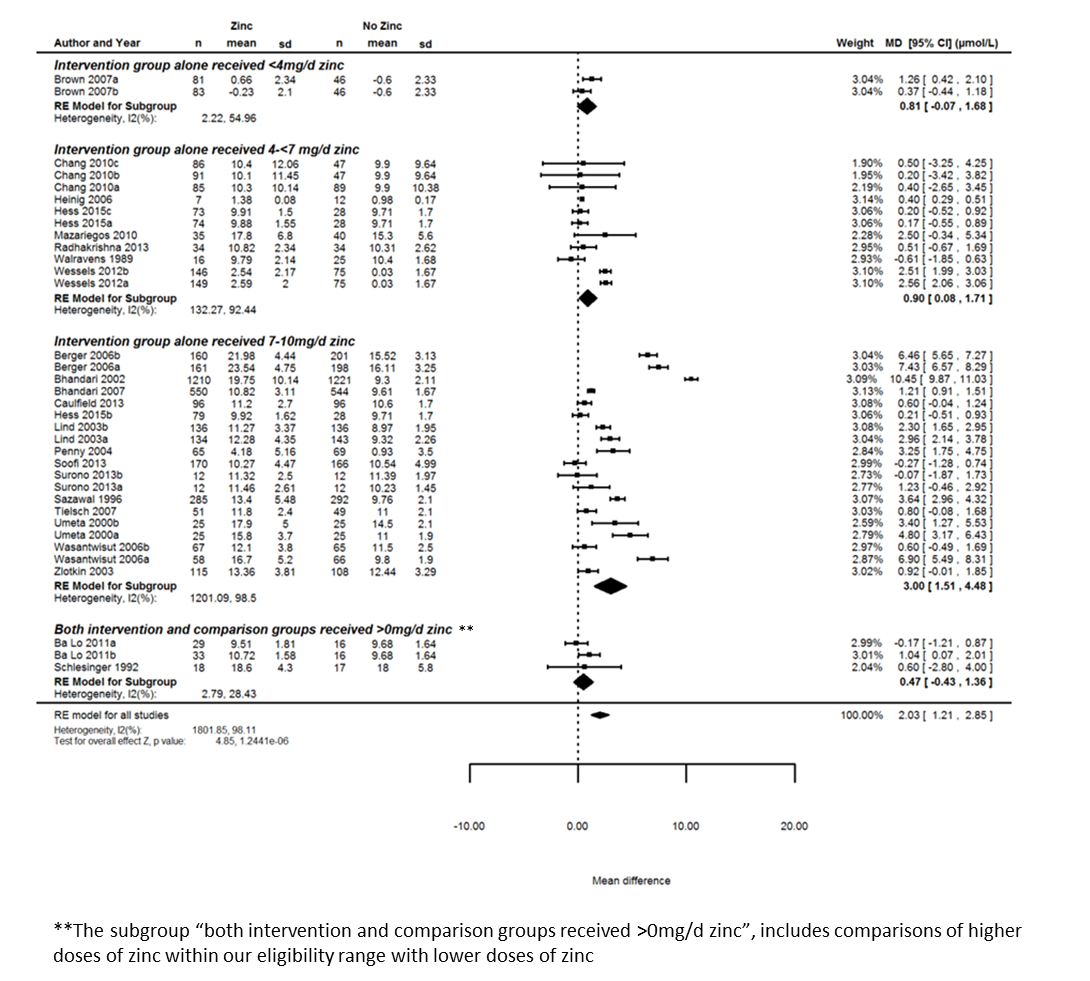


**Figure S28.** Forest plot summarizing the effect of interventions supplying up to 10 mg of additional zinc daily to children 6 to 23 months old, on serum or plasma zinc concentrations, stratified by dose.


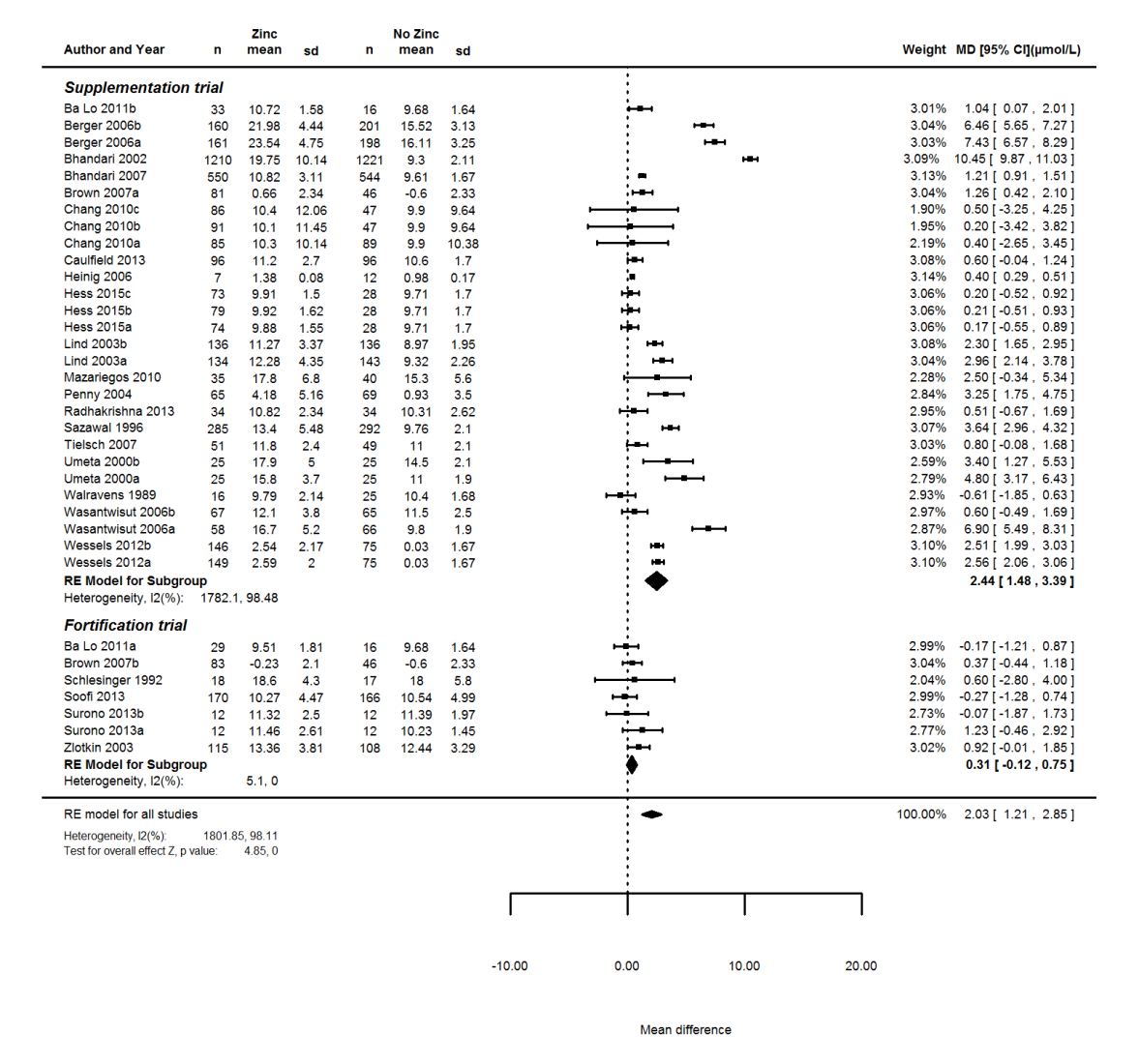


**Figure S29.** Forest plot summarizing the effect of interventions supplying up to 10 mg of additional zinc daily to children 6 to 23 months old, on serum or plasma zinc concentrations, stratified by intervention type.


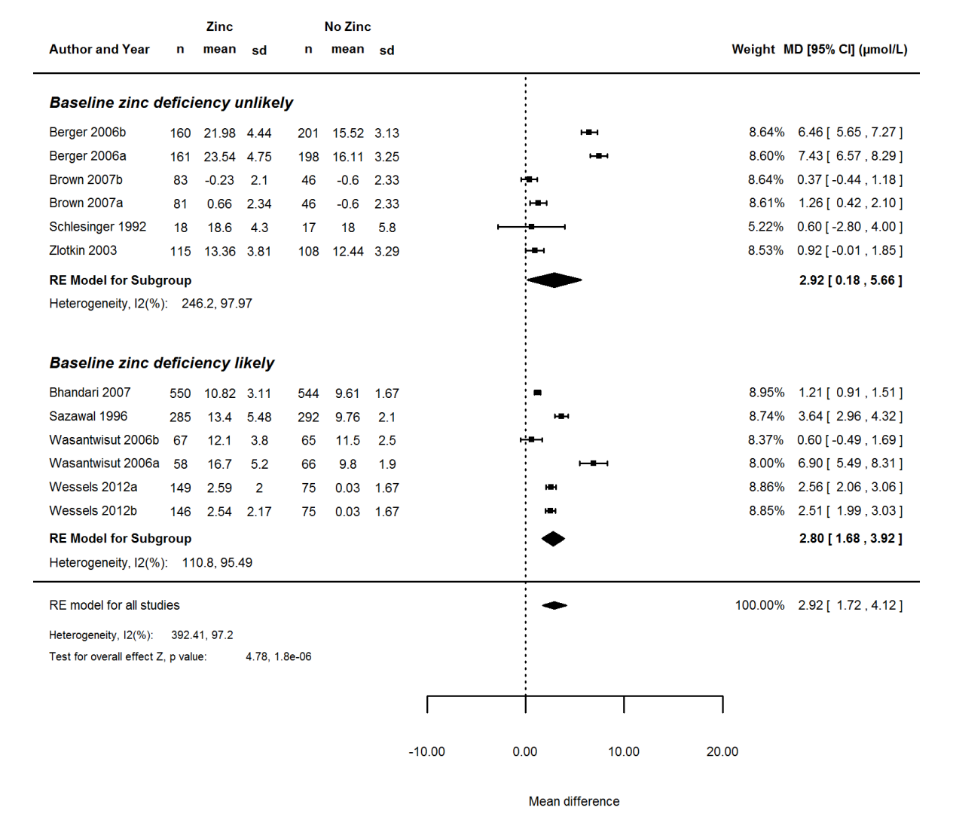


**Figure S30.** Forest plot summarizing the effect of interventions supplying up to 10 mg of additional zinc daily to children 6 to 23 months old on zinc deficiency; analysis stratified by baseline status.


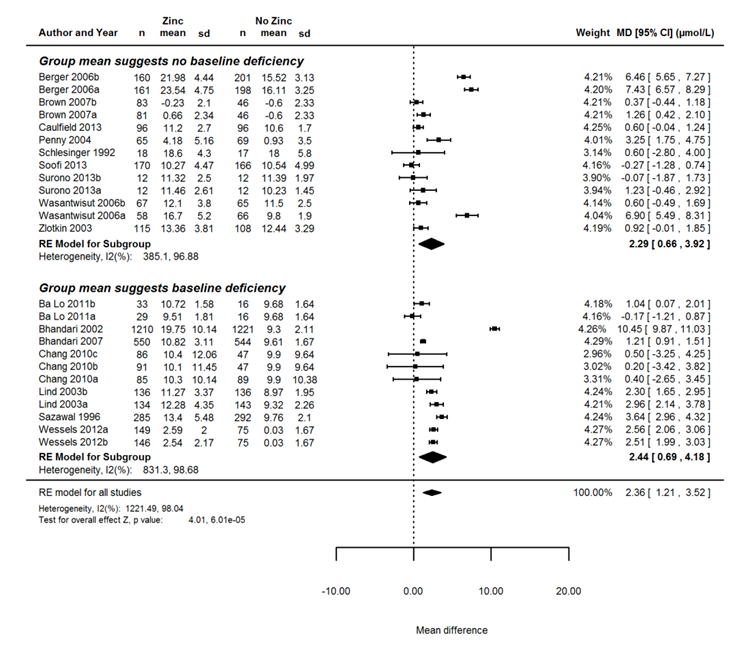


**Figure S31.** Forest plot summarizing the effect of interventions supplying up to 10 mg of additional zinc daily to children 6 to 23 months old on serum zinc; analysis stratified by baseline status.


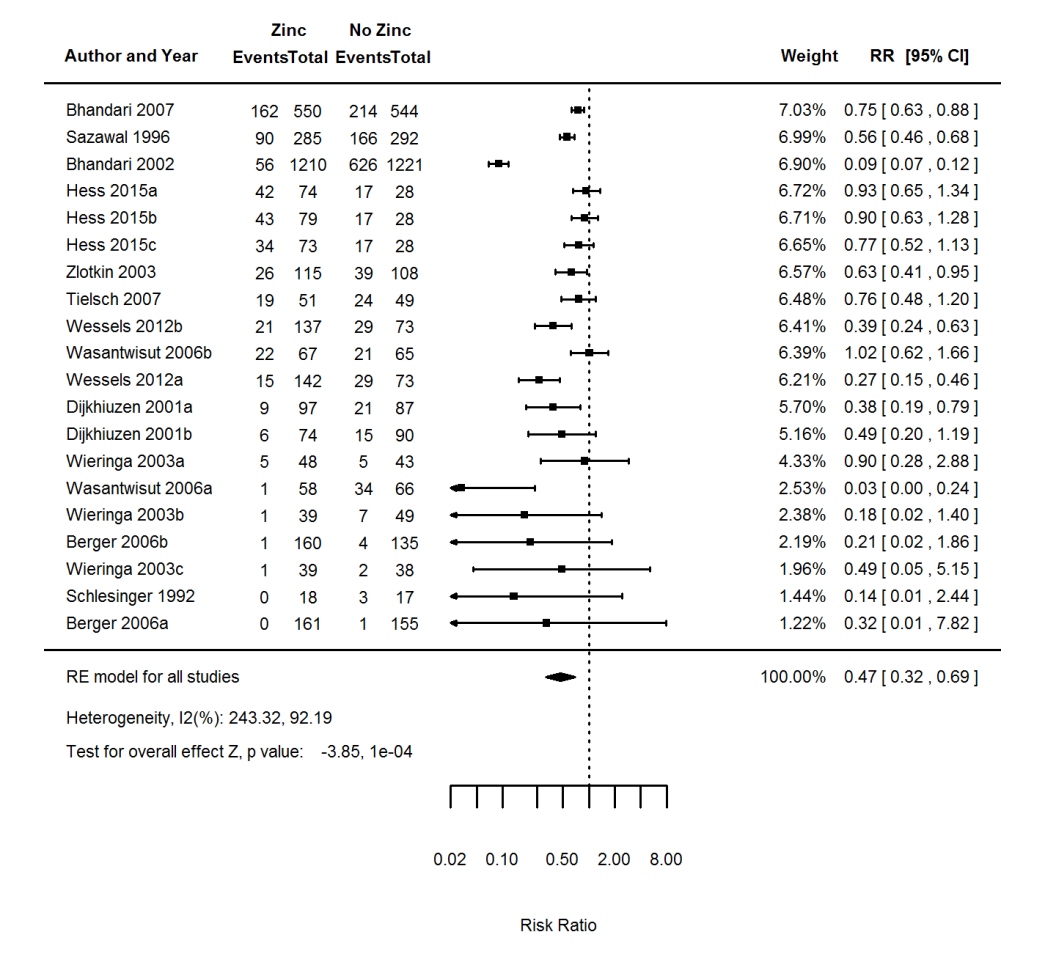


**Figure S32.** Forest plot summarizing the effect of interventions supplying up to 10 mg of additional zinc daily to children 6 to 23 months old, on the risk of zinc deficiency.


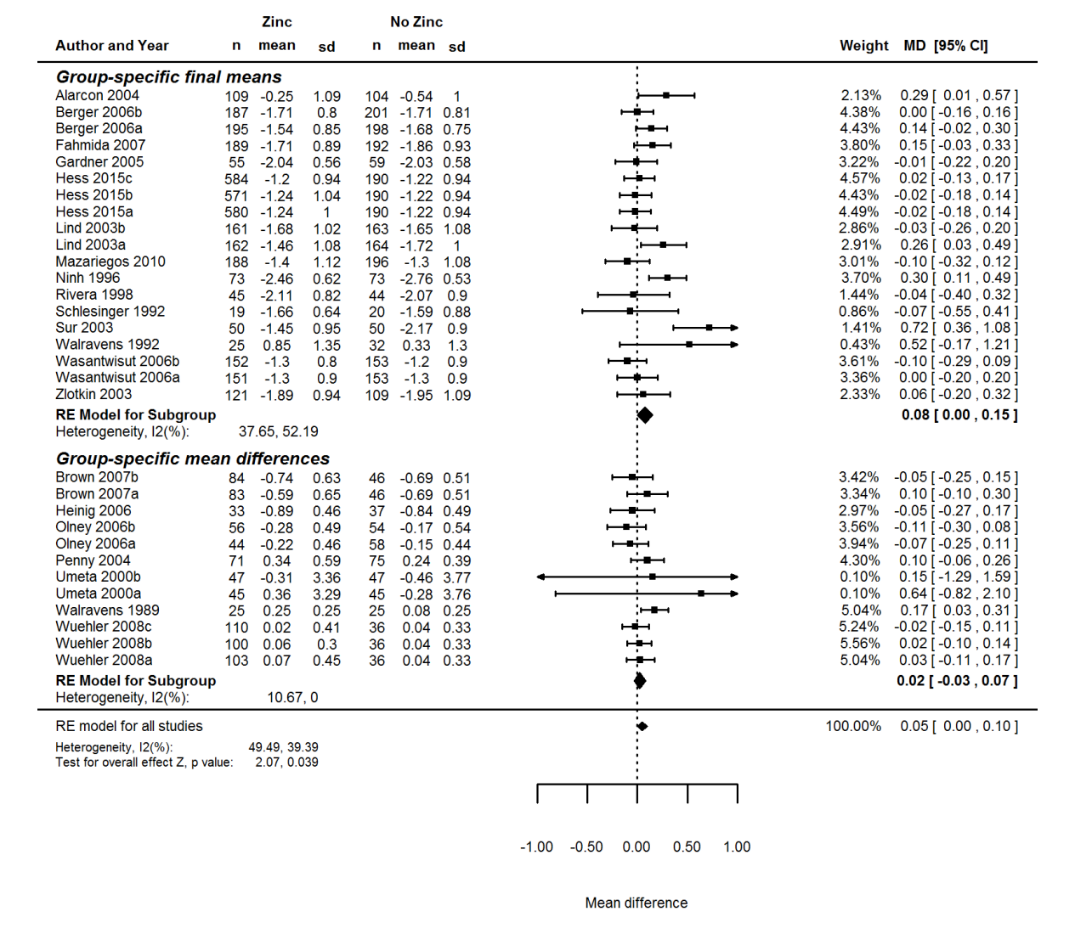


**Figure S33.** Forest plot summarizing the effect of interventions supplying up to 10 mg of additional zinc daily to children 6 to 23 months old on weight-for-age *Z* scores (WAZ).


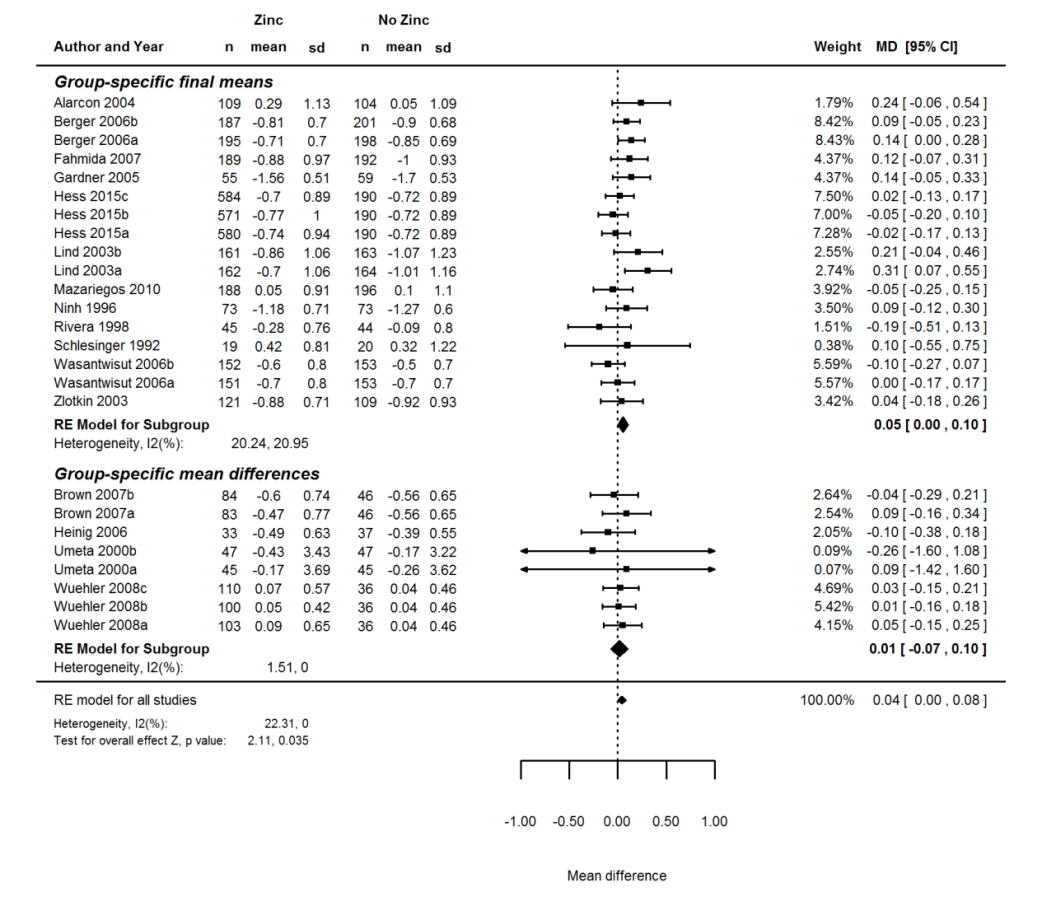


**Figure S34.** Forest plot summarizing the effect of interventions supplying up to 10 mg of additional zinc daily to children 6 to 23 months old on weight-for-height *Z* scores (WHZ).


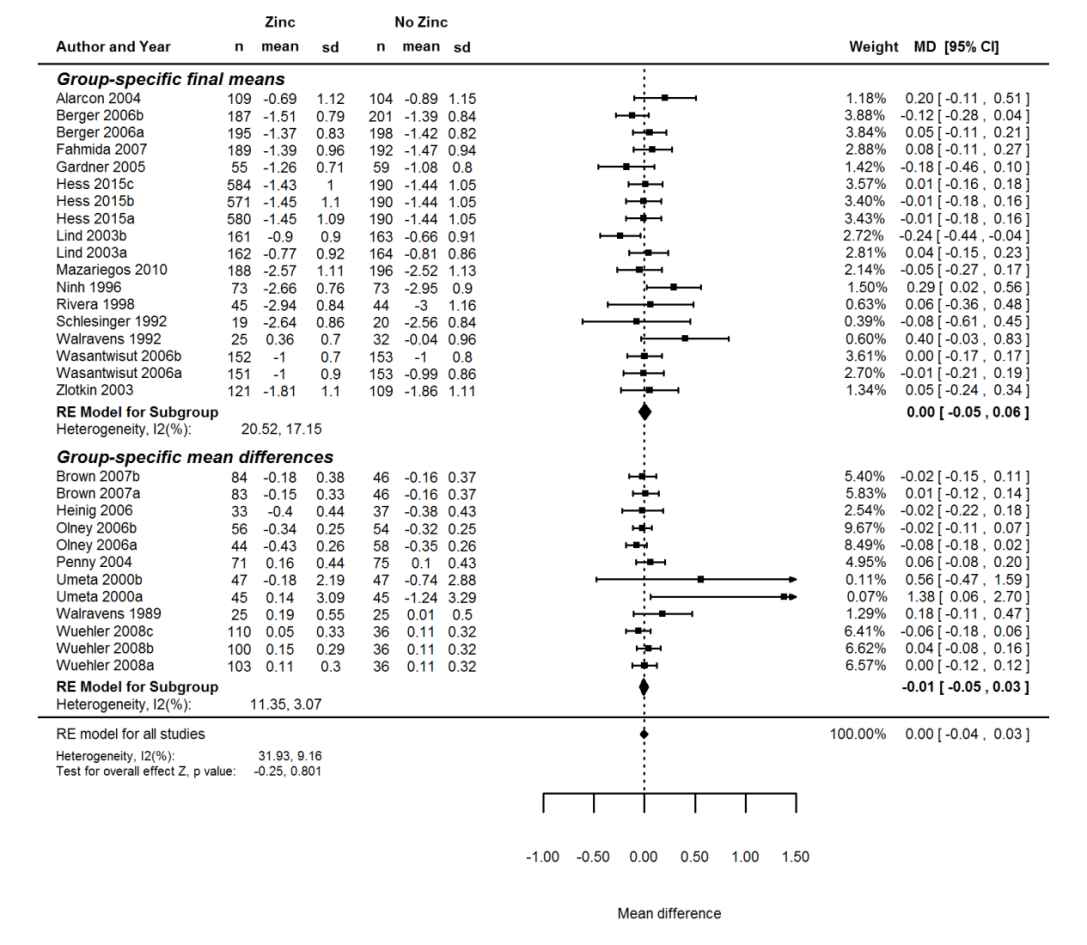


**Figure S35.** Forest plot summarizing the effect of interventions supplying up to 10 mg of additional zinc daily to children 6 to 23 months old on height-for-age *Z* scores (HAZ).


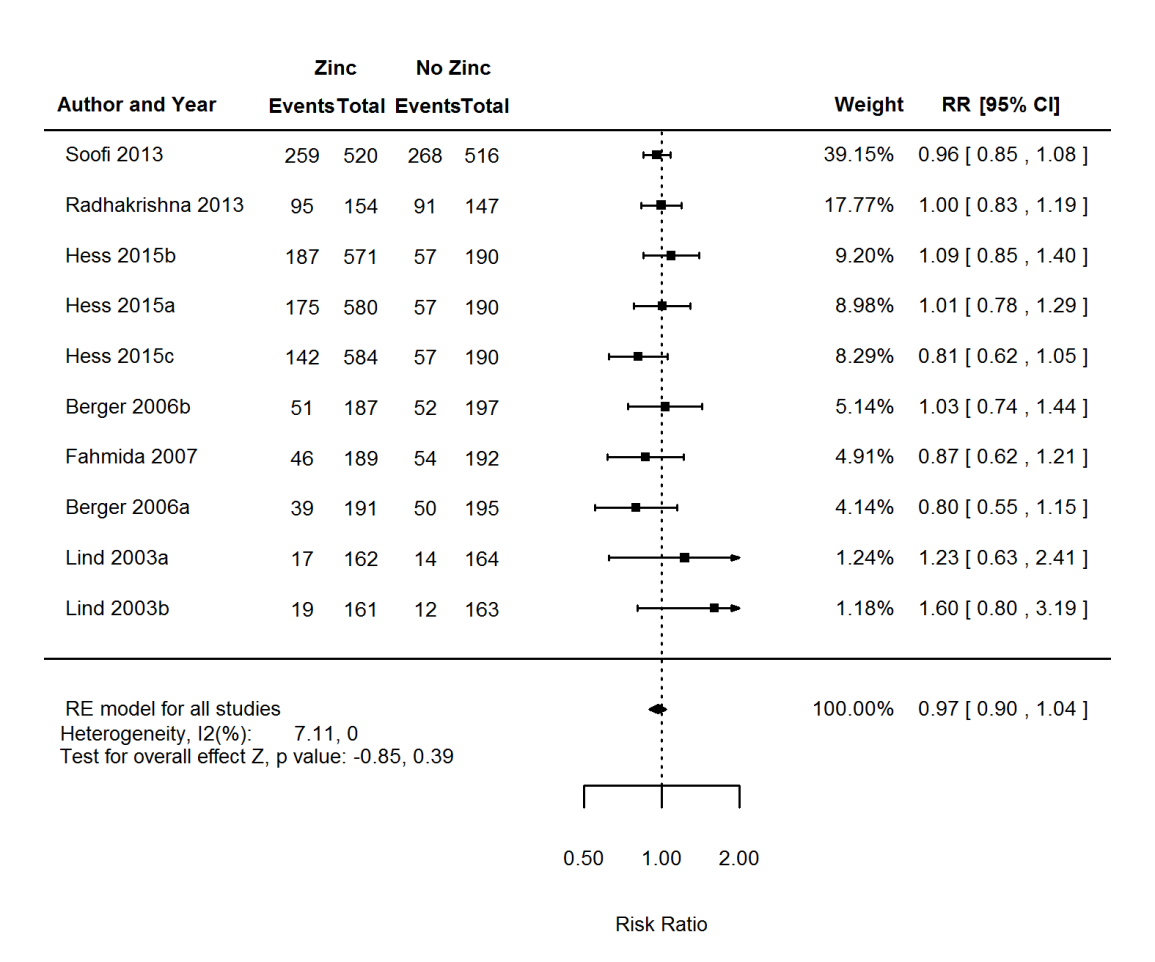


**Figure S36.** Forest plot summarizing the effect of interventions supplying up to 10 mg of additional zinc daily to children 6 to 23 months old on the risk of childhood stunting.


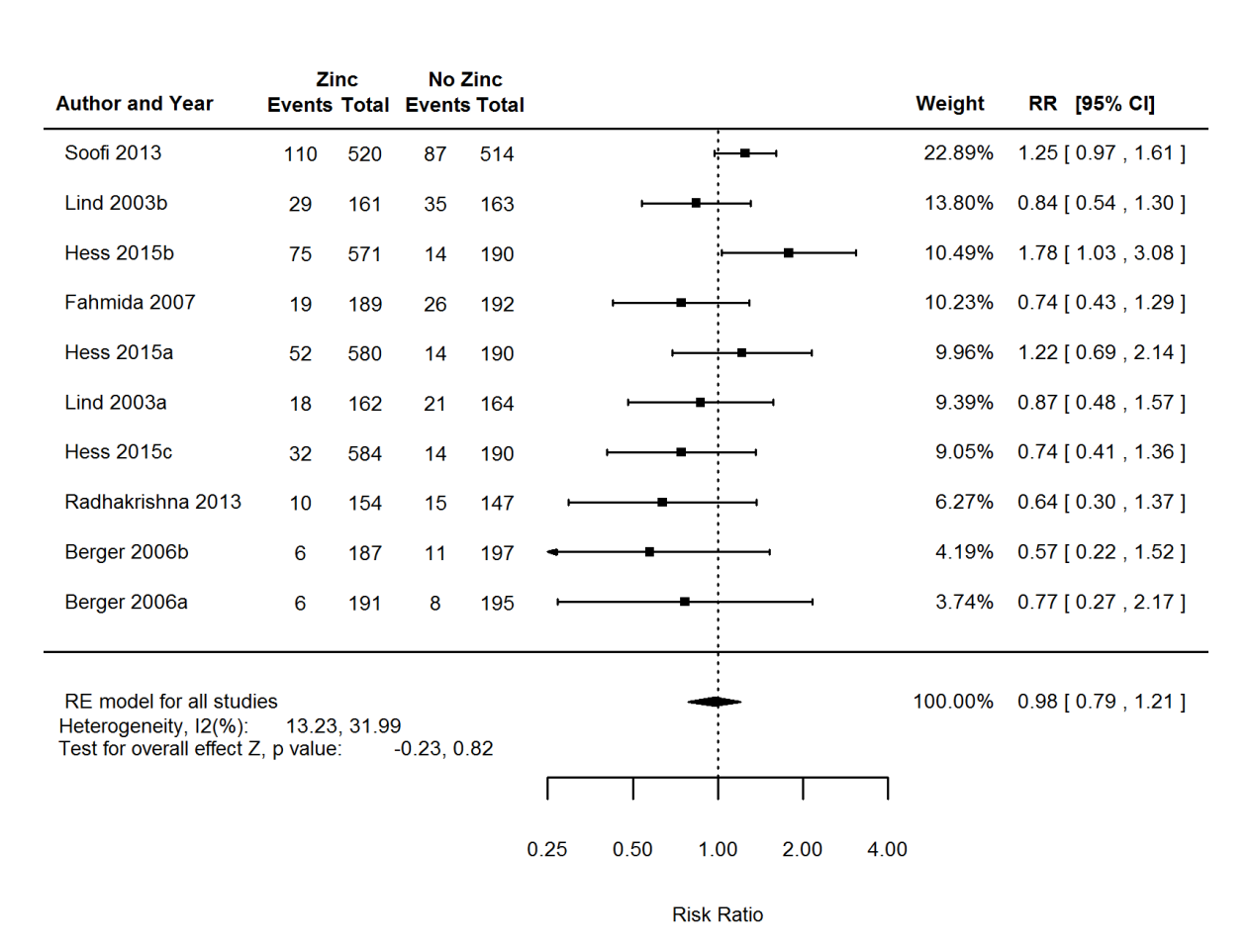


**Figure S37.** Forest plot summarizing the effect of interventions supplying up to 10 mg of additional zinc daily to children 6 to 23 months old on the risk of childhood wasting.


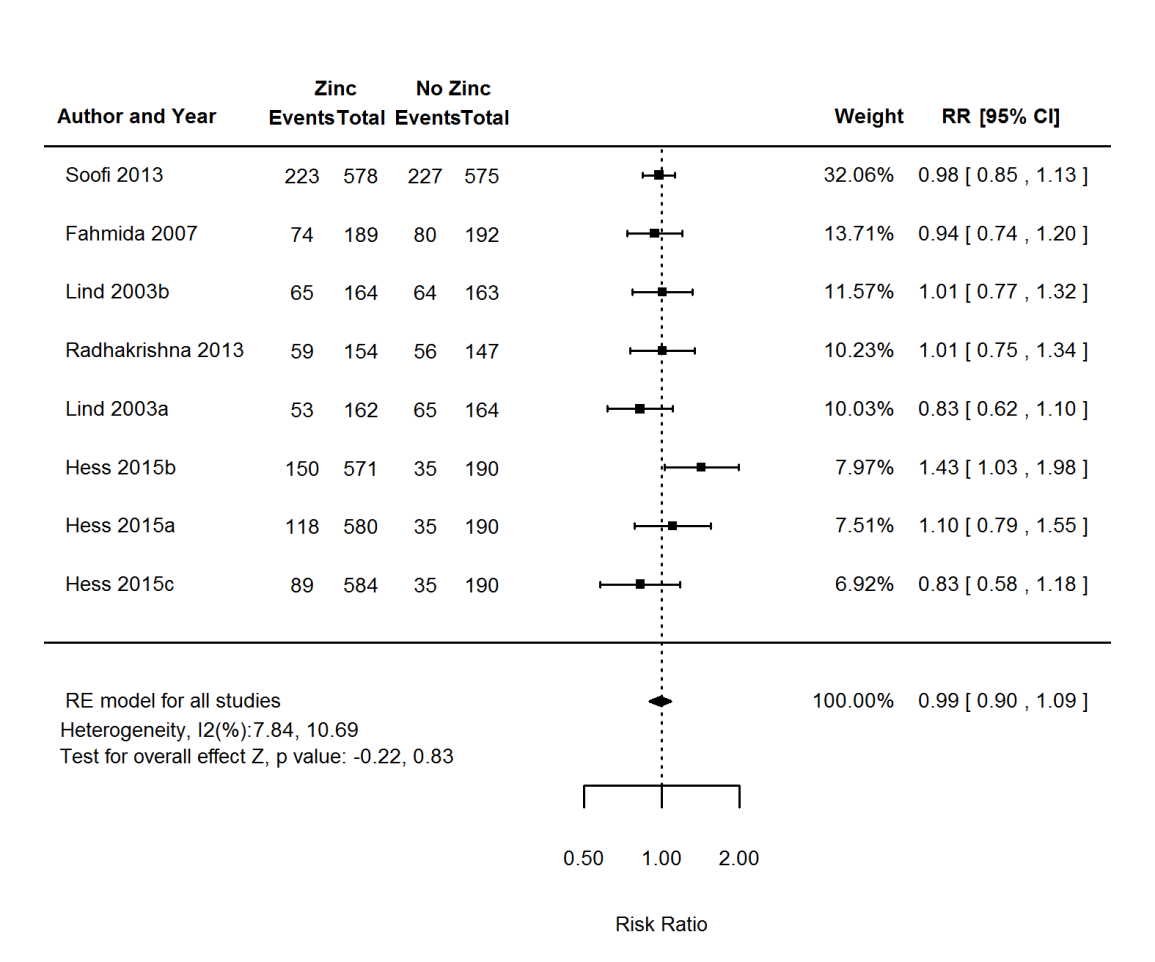


**Figure S38.** Forest plot summarizing the effect of interventions supplying up to 10 mg of additional zinc daily to children 6 to 23 months old on the risk of childhood underweight.

**Table S1.** Quality of evidence (GRADE) summary tables for all outcomes.

| **Outcomes** | **Relative Effect  (95% CI)** | **Studies  (*n*, Participants)** | **Factors Decreasing Quality of Evidence** | **Factors Increasing Quality of Evidence** | **Quality of Evidence (GRADE) ^1^** |
| --- | --- | --- | --- | --- | --- |
| *Iron interventions* | | | | | |
| Birth weight | MD 38 (−16; 91) | 6, (13,627) | Large statistical heterogeneity, imprecision, indirectness |  | Very low |
| Low birth weight | RR 0.69 (0.38; 1.26) | 5, (12,845) | Large statistical heterogeneity, imprecision, lack of studies, indirectness |  | Very low |
| Anemia | RR 0.59 (0.49; 0.70) | 22, (5647) | Large statistical heterogeneity, indirectness |  | Low |
| Hemoglobin (g/L) | MD 4.1 (2.8; 5.3) | 30, (6569) | Large statistical heterogeneity |  | Moderate |
| Iron deficiency | RR 0.22 (0.14; 0.35) | 13, (3698) | Large statistical heterogeneity | Large magnitude of effect | High |
| Iron deficiency anemia | RR 0.20 (0.11; 0.37) | 8, (3464) | Large statistical heterogeneity | Large magnitude of effect | High |
| Iron status using sf concentrations (µg/L) | MD 17.3 (13.5; 21.2) | 21, (4291) | Large statistical heterogeneity | Dose response gradient | High |
| Stunting | RR 1.09 (0.92; 1.29) | 4, (2159) | Lack of studies, indirectness |  | Low |
| Wasting | RR 1.11 (0.84; 1.47) | 4, (1975) | Lack of studies, indirectness |  | Low |
| HAZ | MD −0.02 (−0.08; 0.04) | 10, (3511) | indirectness |  | Moderate |
| WAZ | MD −0.01 (−0.08; 0.05) | 10, (3511) | indirectness |  | Moderate |
| WHZ | MD 0.02 (−0.06; 0.09) | 9, (3297) | indirectness |  | Moderate |
| MDI | MD 0.4 (−0.9; 1.7) | 4, (1062) | Large heterogeneity, lack of studies, indirectness |  | Very Low |
| PDI | MD 0.6 (−1.2; 2.4) | 4, (1062) | Large heterogeneity, indirectness,  lack of studies |  | Very low |
| *Zinc interventions* | | | | | |
| Birth weight | MD 1 (−32; 35) | 8, 3457 | Indirectness |  | Moderate |
| Low birth weight | RR 0.96 (0.67; 1.37) | 6, 2518 | Indirectness |  | Moderate |
| Zinc deficiency | RR 0.47 (0.32; 0.69) | 12, (6666) | Large statistical heterogeneity, indirectness | Large magnitude of effect | Moderate |
| Zinc status using serum zinc concentration (µmol/L) | MD 2.0 (1.2; 2.9) | 23, (8848) | Large statistical heterogeneity, indirectness | Dose response gradient | Moderate |
| Stunting | RR 0.97 (0.90; 1.04) | 6, (5443) | Indirectness |  | Moderate |
| Wasting | RR 0.98 (0.79; 1.21) | 6, (5441) | Indirectness |  | Moderate |
| Underweight | RR 0.99 (0.90; 1.09) | 5, (4793) | Lack of studies, indirectness |  | Low |
| HAZ | MD 0.00 (−0.04; 0.03) | 20, (7340) | Indirectness |  | Moderate |
| WAZ | MD 0.05 (0.00; 0.10) | 21, (7440) | Indirectness |  | Moderate |
| WHZ | MD 0.04 (0.00; 0.08) | 16, (6875) | Indirectness |  | Moderate |

CI, Confidence interval; HAZ, height for age *z*-score; MD, Mean difference; MDI, Mental Development Index; PDI, Psychomotor Development Index; RR: Risk ratio; WAZ, weight for age *z*-score; WHZ, weight for height *z*-score.^1^ GRADE Working Group grades of evidence: High quality: We are very confident that the true effect lies close to that of the estimate of the effect; Moderate quality: We are moderately confident in the effect estimate: The true effect is likely to be close to the estimate of the effect, but there is a possibility that it is substantially different; Low quality: Our confidence in the effect estimate is limited: The true effect may be substantially different from the estimate of the effect; Very low quality: We have very little confidence in the effect estimate: The true effect is likely to be substantially different from the estimate of effect.
